# Systematic review of artificial intelligence use in behavioral analysis of invertebrate and larval model organisms: methods, applications and future recommendations

**DOI:** 10.3389/frai.2026.1789644

**Published:** 2026-05-19

**Authors:** Zuzanna Stępnicka, Natalia Piórkowska, Malwina Brożyna, Tomasz Matys, Adam Junka

**Affiliations:** 1 Department of Translational Technologies, “PUMA”, Platform for Unique Model Applications, Faculty of Pharmacy, Wroclaw Medical University, Wrocław, Poland; 2 Division of Software Engineering and Data Science, Department of Applied Computer Science, Faculty of Information and Communication Technology, Wrocław University of Science and Technology, Wrocław, Poland; 3 The Department and Clinic of Angiology and Internal Medicine, Faculty of Medicine, Wroclaw Medical University, Wrocław, Poland; 4 College of Life Sciences and Medicine, Zhejiang Sci-Tech University, Hangzhou, China

**Keywords:** artificial intelligence, behavioral analysis, deep learning, invertebrates, larva, machine learning

## Abstract

Invertebrate and larval model organisms such as *Drosophila melanogaster*, *Caenorhabditis elegans*, *Danio rerio* larvae, and *Galleria mellonella* are increasingly employed in biomedical, toxicological, and ecological research. Their behavioral responses serve as sensitive indicators of functional changes, yet traditional methods of observation remain low-throughput, subjective, and poorly scalable. Artificial intelligence (AI), including machine learning (ML) and deep learning (DL), has emerged as a powerful alternative, enabling automated and unbiased analysis of highly dimensional behavioral data. Here, we present the first systematic review comprehensively mapping the use of AI in behavioral analysis of invertebrate and larval organisms. Following PRISMA 2020 guidelines, we screened literature published between 2015 and May 2025. A total of 97 eligible studies were analyzed for model organisms investigated, AI methods applied, input data characteristics, preprocessing pipelines, model architectures, and evaluation metrics. We observed a steep increase in publications, from only 2 in 2015 to 97 by mid-2025, with the majority originating from the USA, China, and Germany. The most frequently studied organisms included *D. melanogaster*, *C. elegans*, and zebrafish larvae, alongside aquaculture and pest species. Since 2021, DL models, particularly convolutional neural networks (CNNs), including YOLO models, and pose estimation frameworks such as DeepLabCut have dominated the field, while supervised ML remains common for classification tasks, and unsupervised learning is primarily applied in exploratory clustering. Input data were typically video or image recordings, but reporting practices were highly inconsistent regarding resolution, frame rate, preprocessing steps, and model training details. Evaluation metrics also varied widely, limiting reproducibility and cross-study comparisons. To address these gaps, we propose a standardized reporting framework encompassing input data specifications, preprocessing pipelines, model architecture, and evaluation metrics. Such standardization will enhance transparency, reproducibility, and comparability across laboratories. AI-driven behavioral analysis has the potential to accelerate drug discovery, toxicology, and environmental monitoring while reducing reliance on vertebrate models in preclinical research.

## Introduction

Model organisms, particularly invertebrates such as insects (*Drosophila melanogaster*) and nematodes (*Caenorhabditis elegans*), have become frequently used tools in contemporary biomedical and pharmacological research. These model organisms retain a wide range of evolutionarily conserved biological pathways, including those governing neurodevelopment, innate immunity, stress response, and metabolism, enabling, to a reasonable extent, extrapolations to vertebrates, including humans. For example, *C. elegans* share homologs for up to 60–80% of human disease-related genes, underlining their utility in pharmacological and toxicological modeling (Obafemi et al., 2025). In addition, their short life cycle, ease of handling, low maintenance costs, and minimal or no ethical constraints make them particularly suitable for high-throughput screening platforms (Pastorino et al., 2024).

Larval stages of these invertebrates offer unique experimental advantages over adult forms, particularly in the context of neurobehavioral and toxicological assessment. Their structural and functional simplicity, reflected in reduced tissue and behavioral complexity, minimizes biological noise and facilitates more direct interpretation of experimental outcomes. Larval organisms often demonstrate high physiological sensitivity to chemical stimuli, making them ideal biosensors for detecting low-dose effects and early biomarkers of toxicity (Strähle et al., 2012). Metrics such as lethal dose 50 (LD₅₀), no observed effect concentration (NOEC), or changes in specific behavioral repertoires (e.g., exploratory behavior, locomotion, phototaxis) are frequently used to quantify toxic or pharmacological effects. For instance, *Danio rerio* larvae show quantifiable locomotor or startle responses to anxiolytics, antiepileptics, and neurotoxins (Sison and Gerlai, 2011). *C. elegans* larvae have been instrumental in dissecting dopaminergic neurodegeneration and behavioral plasticity in response to diverse pharmacological compounds (Smith et al., 2019a). More recently, *Galleria mellonella* larvae have gained traction as a cost-effective alternative *in vivo* model in infection biology, immunotoxicology, and behavioral pharmacology. Their innate immune system shares several features with that of mammals, including conserved pathways for phagocytosis, production of reactive oxygen species (ROS), and synthesis of antimicrobial peptides (AMP). Unlike many invertebrate models, *G. mellonella* larvae can be maintained at 37 °C (Ménard et al., 2021), which enables direct co-cultivation with human pathogens and exposure to human-derived biofluids or experimental agents. From a behavioral perspective, *G. mellonella* larvae exhibit distinct and measurable changes in posture, locomotion, and escape behavior under various stress conditions, including infection, inflammation, and chemical exposure, which can serve as functional readouts (Ménard et al., 2021; Marena et al., 2025). It positions *G. mellonella* as a promising model at the intersection of toxicology, translational medicine, and behavioral neuroscience (Asai et al., 2023).

Behavioral analysis has become one of the key approaches in preclinical and translational research, offering means of assessing the effects of pharmacological agents, neurotoxicants, and environmental stressors. Unlike morphological or molecular markers, which often capture static or endpoint changes, behavior reflects the dynamic output of interacting physiological systems, including the nervous, muscular, and endocrine networks (Ratner and Farb, 2022). As such, it can reveal functional alterations, long before structural damage becomes evident, making it a particularly valuable early indicator of adverse or therapeutic effects. In invertebrate models, behavior is increasingly employed to quantify neurophysiological integrity and systemic health. Parameters such as total distance traveled, swim velocity, angular displacement, turn frequency, freezing time, and burst activity are routinely extracted from these model organisms. For instance, such locomotor traces of zebrafish larvae are used to investigate their response to drugs targeting GABAergic (gamma-aminobutric acid-ergic), glutamatergic, or dopaminergic pathways (Rosa et al., 2022). The exposure to anxiolytics typically reduces thigmotaxis and increases center zone activity, while proconvulsants induce erratic movement or seizure-like bursting (Maximino et al., 2010). In *C. elegans*, chemotaxis assays, omega turns, and body bends are used to probe sensory integration and motor coordination, while in *Steinernema carpocapsae* nematode host-seeking behavior is explored (Manduca et al., 2025). These behaviors are tightly regulated by conserved neurotransmitter systems and have been validated against known neuroactive compounds. Beyond classic dose–response studies, behavioral phenotyping is being leveraged in phenotypic screening pipelines and complex toxicity profiling. For instance, behavioral changes can serve as functional proxies for neurotoxicity, mitochondrial dysfunction, or developmental delay, especially when other biomarkers are inconclusive (Jarema et al., 2022).

The invertebrates’ behavior, especially in larval form, is also highly responsive to environmental toxins, food additives, and biological fluids, making it applicable in ecotoxicology, forensics, and environmental monitoring. Functional endpoints like latency to stimulus response or recovery time after exposure have been proposed as alternative metrics for evaluating sublethal effects in regulatory toxicology frameworks (Jiang et al., 2016). Importantly, behavioral endpoints offer translational relevance. Phenotypes such as hypoactivity, hyperactivity, ataxia, startle failure, or habituation deficits often mirror clinical symptoms in neurological and psychiatric disorders. As such, larval behavior may be perceived not just as a readout of gross toxicity, but as a correlate of cognitive, emotional, or neuromotor function. Moreover, because behavior can be continuously and non-invasively monitored, it is well-suited for longitudinal studies, dynamic dose modeling, and real-time phenotypic screening. In summary, behavioral analysis in invertebrate and larval organisms may bridge the gap between molecular changes and organism-level function, enabling high-sensitivity evaluation of pharmacological and toxicological effects in a scalable manner.

Traditionally, behavioral analysis relied on two main mechanisms. Either behavior was observed by a qualified researcher, or an assay was designed to measure a specific aspect of behavior (Egnor and Branson, 2025). For instance, human-based scoring of number of courtship behaviors in genetically mutated fruit flies showed that single gene splicing can specify complex innate behavior (Demir and Dickson, 2005). Nevertheless, such traditional approaches are time-consuming, low-throughput and prone to observer bias. Moreover, they are particularly inadequate for detecting subtle or transient phenotypes, especially those involving complex temporal dynamics or multi-dimensional movement patterns (Desland et al., 2014). Semi-automated tracking systems have improved the throughput and objectivity of behavioral studies but still depend heavily on human input. These systems typically extract positional data (e.g., centroid tracking, velocity, trajectory curvature) from video recordings, and provide derived metrics such as distance traveled, acceleration, or turning angles. For instance, locomotion on *D. rerio* in the larval stage, treated with early-stage therapeutics, was validated as an early-stage toxicity screen (Winter et al., 2008). Undoubtedly, being an improvement on traditional behavioral analysis, these methods often struggle with occlusions, low contrast, complex postures, or with overlapping individuals in group settings. Moreover, predefined rule-based classifications (e.g., “freezing” if speed reduces to a predefined value) may oversimplify complex and/or ambiguous behaviors. Importantly, both manual and semi-automated methods lack, to the major extent, scalability and adaptability (Luxem et al., 2022). Behavioral experiments generate large volumes of video data, yet traditional tools are ill-equipped to handle these datasets at scale.

Building upon traditional and semi-automated approaches, recent advances in artificial intelligence (AI) have begun to transform behavioral analysis landscape. Ranging from organisms’ automatic detection, classification, thorough pose estimation, tracking and behavior clustering AI-based techniques revolutionized the field of behavioral biology. For instance, detection tools can accurately distinguish pests’ species for agricultural purposes (Tannous et al., 2023). Machine learning (ML), which is a core branch of AI, deals with programming computers to “learn” patterns in data. It can be done by providing labeled data (e.g., images) to the algorithm [referred to as supervised learning (SL)] or by automatically grouping data into clusters of alike characteristics without training the model on predefined labeled data [referred to as unsupervised learning (UL)]. These models are relatively straight-forward to build, work well on smaller, preselected datasets and in general are easily interpretable. On the other hand, they still rely on human input at the model training stage, selecting which behavioral parameters should be considered by the model-called feature selection. A dynamically growing group of deep learning (DL) approaches aims at tackling some of the limitations of classical ML. This group includes the most popular convolutional neural networks (CNN), recurrent neural networks (RNN), and Transformers, among others. These models take a more flexible and objective approach to feature selection and work on highly dimensional and unstructured data. Unfortunately, this comes at the cost of the requirement for a significantly larger training dataset and less transparent and reproducible methodology. It is important to clarify that both ML and DL are subsets of AI, distinguished primary by their model architecture. In contrast, SL and UL refer to learning paradigms (strategies by which models are trained). Accordingly, when restricting the scope of AI to behavioral analysis of invertebrate and larval model organisms, we categorize AI-based approaches into supervised DL (sDL), supervised ML (sML), unsupervised ML (uML).

Building upon this emerging potential, it becomes essential to delineate the concrete tools that operationalize these advances for experimental biology. A growing ecosystem of open-source platforms now provides biologists with accessible solutions for pose estimation, multi-animal tracking, and behavior classification. While originally developed within computational sciences, many of these frameworks have been adapted to laboratory contexts, lowering the technical threshold for their use. Table 1 introduces the most prominent of these tools, such as DeepLabCut, SLEAP, and JAABA presenting their application in behavioral analysis.

**Table 1:** Summary of the key AI tools developed for or used in behavioral analysis of invertebrate and larval model organisms.

AI tool	Description	Precursors
DeepLabCut	Open-source software package for markerless 2D and 3D pose estimation of animals. Based on transfer learning with CNN model achieves excellent results with little training data.	none
SLEAP	Open-source package for general-purpose, multi-animal pose tracking. CNN model with pretrained wights, but can also enable transfer learning.	LEAP
FLLIT	FLLIT stands for Feature Learning Leg Segmentation and Tracking. It is a graphical user interface (GUI)-enabled program, MATLAB compatible. Analyzes gait parameters (incl. lurching steps, short strides).	none
WormPose	Open-source package. CNN-based model for estimating challenging poses of *C. elegans*, in 2D. Generates synthetic worm-real worm pair for training.	none
StrongSORT	Multi-object tracking algoritm.	DeepSORT
Z-LAP Tracker	Zebrafish Larvae Position Tracker. Modification of DeepLabCut, specifically trained for zebrafish larvae. Quantifies behavioral parameters.	none
JAABA	Open-source program. Annotate and classify specific behaviors based on kinetic features.	none
Stytra	Open-source tool for tracking, visual simulations and close-up experiments. It is a graphical user interface (GUI)-enabled software shown to work in conjunction with, e.g., DeepLabCut in behavioral experiment.	none
Anipose	Open-source python toolkit for 3D markerless pose estimation, built on 2D pose estimation tool DeepLabCut.	none

These AI tools, along with the fast-evolving field of AI-enabled analysis undoubtedly transformed how researchers study behavior. This interdisciplinary field of biology brings together expertise from computer science, neuroscience, microbiology to ecology, enabling sophisticated approaches to capture non-linear dynamics and complexities of behavior that were previously difficult to quantify or even detect. Despite the growing interest and potential, a survey of the literature reveals that, out of eight identified reviews (Berman, 2018; Adjé et al., 2023; Egnor and Branson, 2025; Robie et al., 2017; Moulin et al., 2021; Mathis and Mathis, 2020; Lima et al., 2020; Pereira et al., 2020), none comprehensively maps the current landscape and critically evaluates the methodological advancements. Consequently, the field suffers from fragmented information, with no clear guidelines for applying these tools across diverse invertebrate and larval models.

This systematic review aims to fill that gap by summarizing the state of AI-based behavioral analysis in these organisms, highlighting both the limitations of current methodologies and the opportunities they present, and providing a forward-looking perspective on their potential translational impact in biological research.

## Methods

This systematic review was conducted in accordance with the Preferred Reporting Items for Systematic Reviews and Meta-Analyses (PRISMA) 2020 guidelines (Page et al., 2020). Its aim was to identify recently published scientific literature on AI methods used in behavioral analysis of invertebrate and/or larval model organisms, summarize current trends in research, and propose future directions. The following subsections present detailed methodology.

### Search strategy

To conduct thorough and systematic search of scientific literature, the Sample, Phenomenon of Interest, Design, Evaluation, Research type (SPIDER) tool was implemented (Cooke et al., 2012). Information summarized in Table 2 aided in formulating database search queries (Supplementary Table S1) and inclusion/exclusion criteria (Table 3).

**Table 2:** The overview of the SPIDER tool used to formulate search queries for the systematic review.

Sample	Invertebrate and larval model organisms.
Phenomenon of Interest	Use of AI methods for behavioral analysis, including detection.
Design	Published literature, including preprints, that included sufficient methodology to understand and evaluate AI models. Studies where behavioral data was acquired via video, image, or motion tracking systems.
Evaluation	Eligible studies report the following: organism model investigated, type of AI-model used, type and quantity of input data, source of input data, preprocessing and analysis steps, where applicable model architecture, and model evaluation metrics.
Research Type	Quantitative, exploratory, and comparative studies.

**Table 3:** Inclusion/exclusion criteria applied during the search procedure in the systematic review.

Inclusion criteria	Exclusion criteria
Stage 1: Initial screening of title and abstracts	
Both empirical studies and literature reviews that used AI-based methods	
Studies investigating behavioral analysis including detection, movement, activity patters, social interactions	
Studies investigating invertebrate and/or organisms in larval stage	Studies focusing entirely on adult, vertebrate organisms
Studies where organisms and their behavior was recorded as images, videos or motion trajectories	
Published between 01.01.2015-31.05.2025	
Full text available in English	
Preprints	Publication type including bachelor’s, master’s and doctorate thesis and book chapters
Stage 2: Full text analysis	
	Studies using only traditional analytical methods to study behavior in model organisms
	Studies recording behavioral analysis via a microscope. Authors define microscope as a static device using series of magnifying lenses to obtain an image or video in a macro scale
	Studies with insufficiently described methodology and results, making it impossible to answer the stated RQs

Furthermore, the following Research Questions (RQ) were identified and used to guide the feature extraction and analysis stages, ensuring a comprehensive and detailed overview.

*RQ1*: What is the current landscape of AI-based research in behavioral analysis of invertebrate and/or larval organisms? This question investigates model organisms used, types of AI models, their applications and overall scientific interest in the field.

*RQ2*: What types of input data and behavioral features are used in these models? This question aims to assess how behavior is recorded and quantified.

*RQ3*: How effective and readable are AI-based models and their analysis pipelines? This question explores preprocessing steps, model architecture (where applicable), and evaluation metrics.

### Search procedure

The search was completed on 31st May 2025, and included literature published between 1st January 2015 and 31st May 2025. This period was chosen to accurately represent the current landscape of the dynamically developing field.

PubMed, Scopus, Web of Science, IEEE Xplore, and ACM Digital Library databases were searched, as per defined queries (Supplementary Table S1). Additionally, to saturate the search, the first 200 publications from Google Scholar search engine were added. The literature was aggregated in Mendeley.

The inclusion criteria were tailored to identify research that investigated behavioral analysis using computational approaches, including image processing pipelines, simple ML algorithms, DL methods, and other AI-based approaches. The studies had to focus, or at least test algorithms on invertebrate organisms or organisms in the larval stage. Moreover, to broaden the scope of analyzed publications, authors decided to include papers which used AI models to investigate behavior (including movement, activity patterns or social interactions) or perform organism’s detection. The organism had to be recorded via a camera or motion sensor, and not with a microscope. This approach ensured that recording sources focused on whole organisms, rather than sub-parts or cells, and downstream analysis of methods could be compared across research. Finally, both peer-reviewed literature and preprints, published between 1st January 2015 and 31st May 2025 were included.

The main exclusion criteria included analyzed behavior using only traditional analytical tools or did not analyze behavior at all. Studies that focused exclusively on adult vertebrates were also excluded. Finally, studies that met inclusion criteria, but did not contain sufficient methodological details on model used; its evaluation metrics, source, type and quantity of input data were also excluded. This ensured both that only studies of high quality were included, and that authors could compare models and their characteristics across studies.

**Figure 1:**
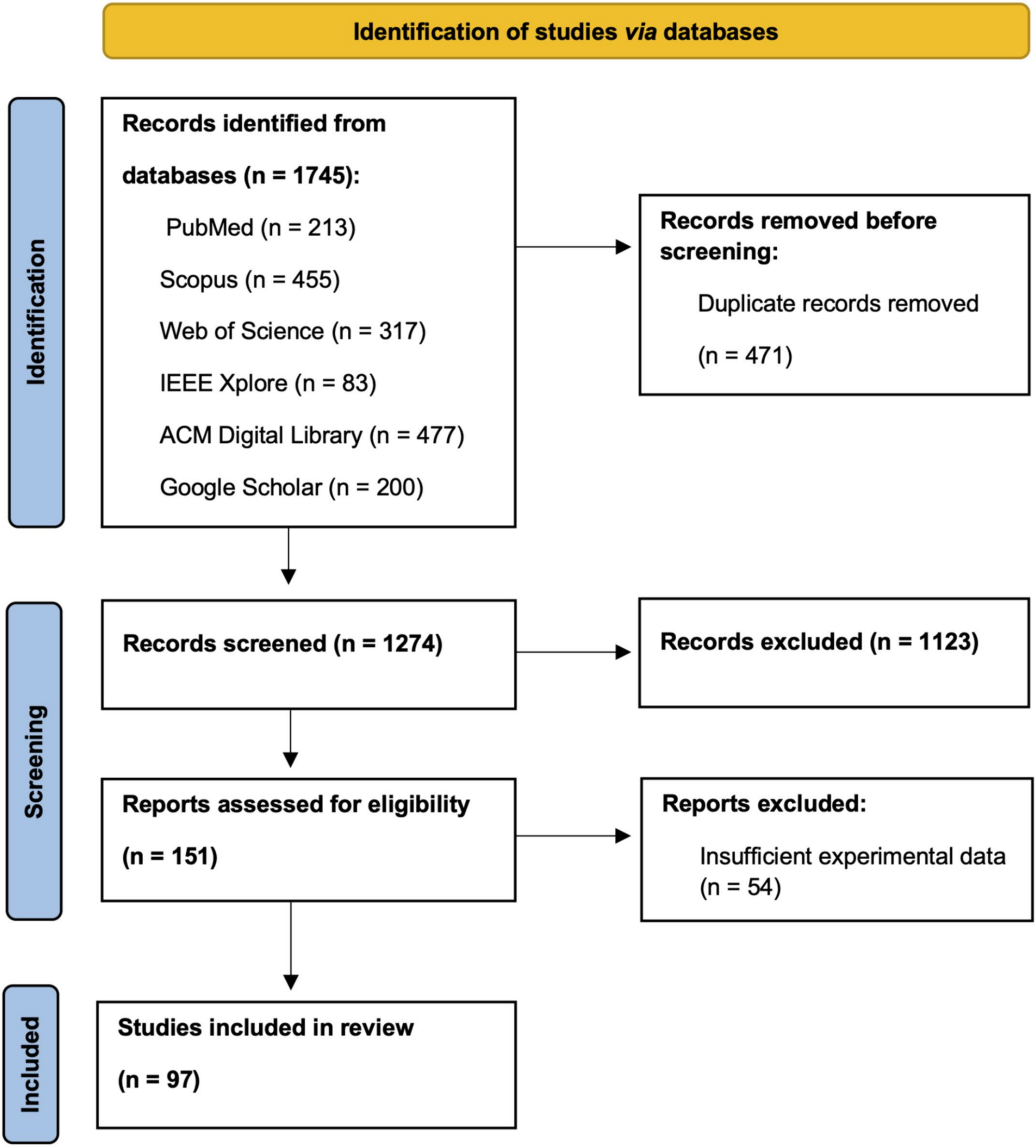
The PRISMA diagram showing systematic search procedure.

The initial database search yielded 1745 results. Out of these records, using Mendeley, 471 duplicates were identified, manually verified and removed. Next, in the first stage of selection, authors screened papers based on titles and abstracts. At this stage, 1,123 records were removed. The remaining 151 publications underwent thorough analysis based on full text. A total 54 of these were further excluded. The literature search was done independently by two authors and, where necessary, discrepancies were resolved through discussion and consultation with the rest of the authors. Below, the PRISMA diagram (Page et al., 2020) summarizes the search procedure (Figure 1).

### Features extraction

The literature included in the final analysis underwent a detailed feature extraction process. Authors decided on a set of key information, relevant to comprehensively answer previously stated research questions. These include:

1. Publication details, publishing journal, publication year, model organism investigated, AI model used, model’s task and aim of the analysis;
2. Type and quantity of input data, source of input data, behavioral characteristics of organism analyzed;
3. Preprocessing steps, model architecture (where applicable), evaluation metrics reported.

Data extraction was carried out by two authors and cross-checked to mitigate extraction errors. The full set containing all extracted data is available in Supplementary Tables S2–S4)

### Data analysis

Aggregated results were summarized in figures and supported by descriptive statistics were applicable. Data was aggregated in Excel, preprocessed and visualized using RStudio (R version 4.5.0). The results were structured around research questions and authors formulated qualitative assessment of methodological rigor.

When studies investigated multiple model organisms, employed hybrid models (meaning more than one model was used) or different types of input data, each instance was indexed and reported separately. Exemption to the rule were studies which compared multiple models. In those cases, only the model evaluated as the best performer was reported. As a result, totals for some statistical comparisons may exceed the total number of analyzed studies. For variables like a country of study and research area investigated, only the primary/main instance was considered. Finally, for variables such as preprocessing steps, model architectures, and evaluation metrics, key information was summarized, with data grouped and reported selectively to highlight elements most relevant to the analysis. Authors decided on this approach to present extracted results in the most informative format, providing a comprehensive landscape of AI methods utilized in behavioral analysis of invertebrate and larval model organisms.

## Results

### Temporal and geographical trends

**Figure 2:**
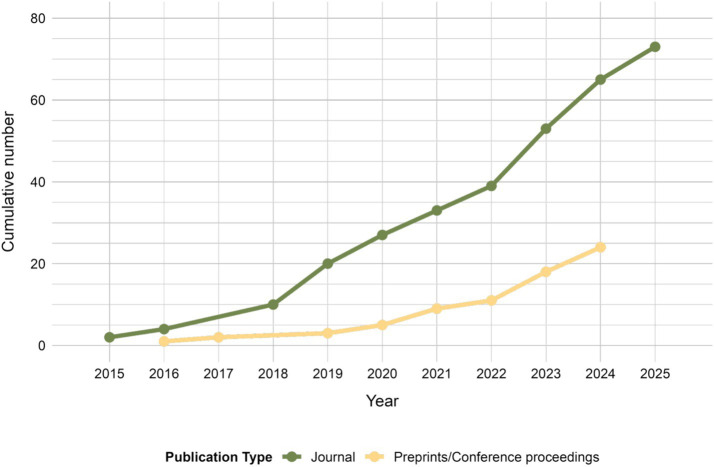
Cumulative number of research published between 2015 and 2025.

**Figure 3:**
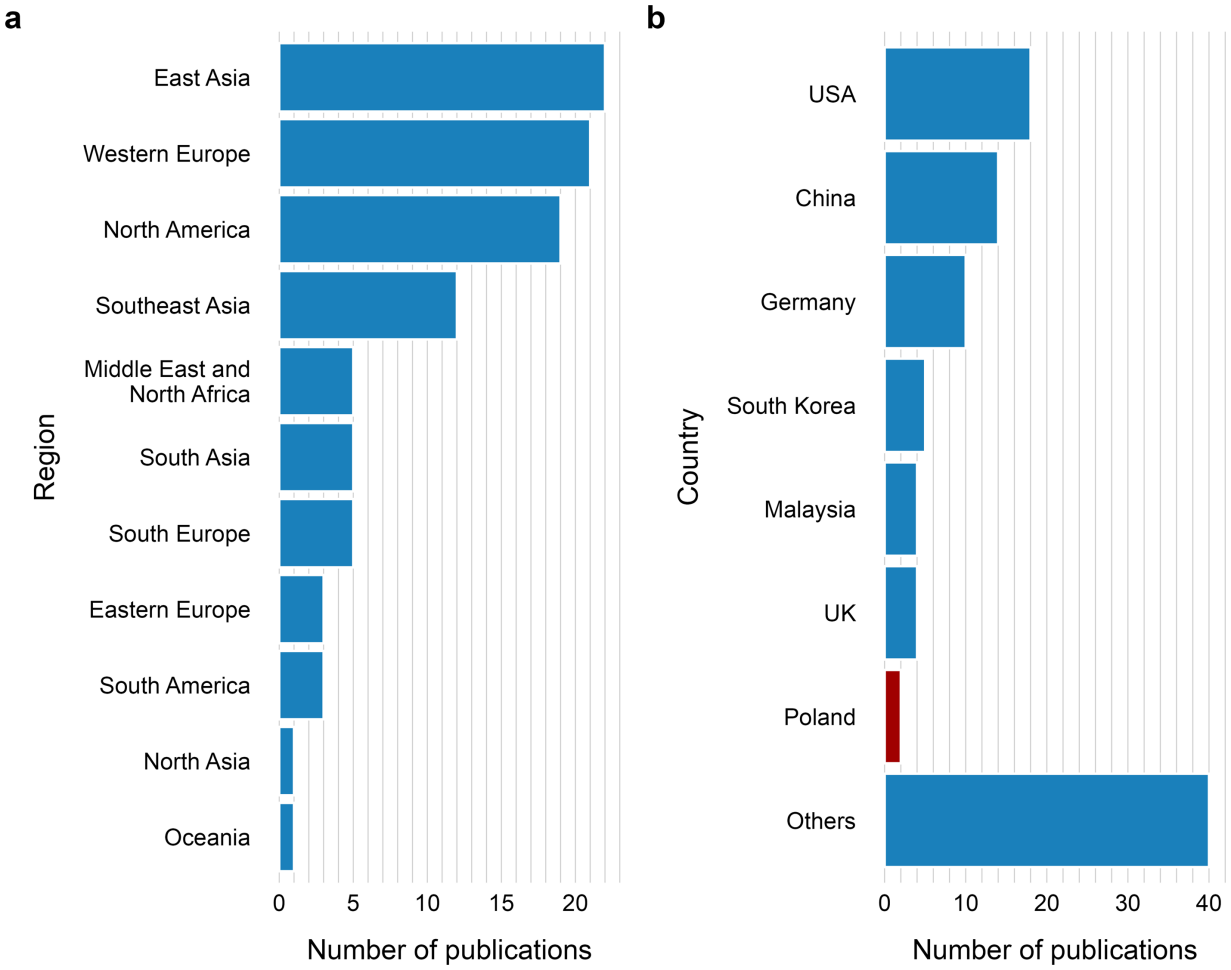
Number of peer-reviewed articles, preprints, and conference proceedings published between 2015–2025, shown by geographical region **(a)** and by country **(b)**. Panel **(a)** presents a complete regional distribution. Panel **(b)** highlights the most productive countries, while all remaining countries are grouped as “Others” to preserve clarity and avoid over fragmentation. This grouping also illustrates that the combined contribution of all remaining counters is comparable to that of the leading contributors. The country of authors (Poland) is indicated in red.

**Figure 4:**
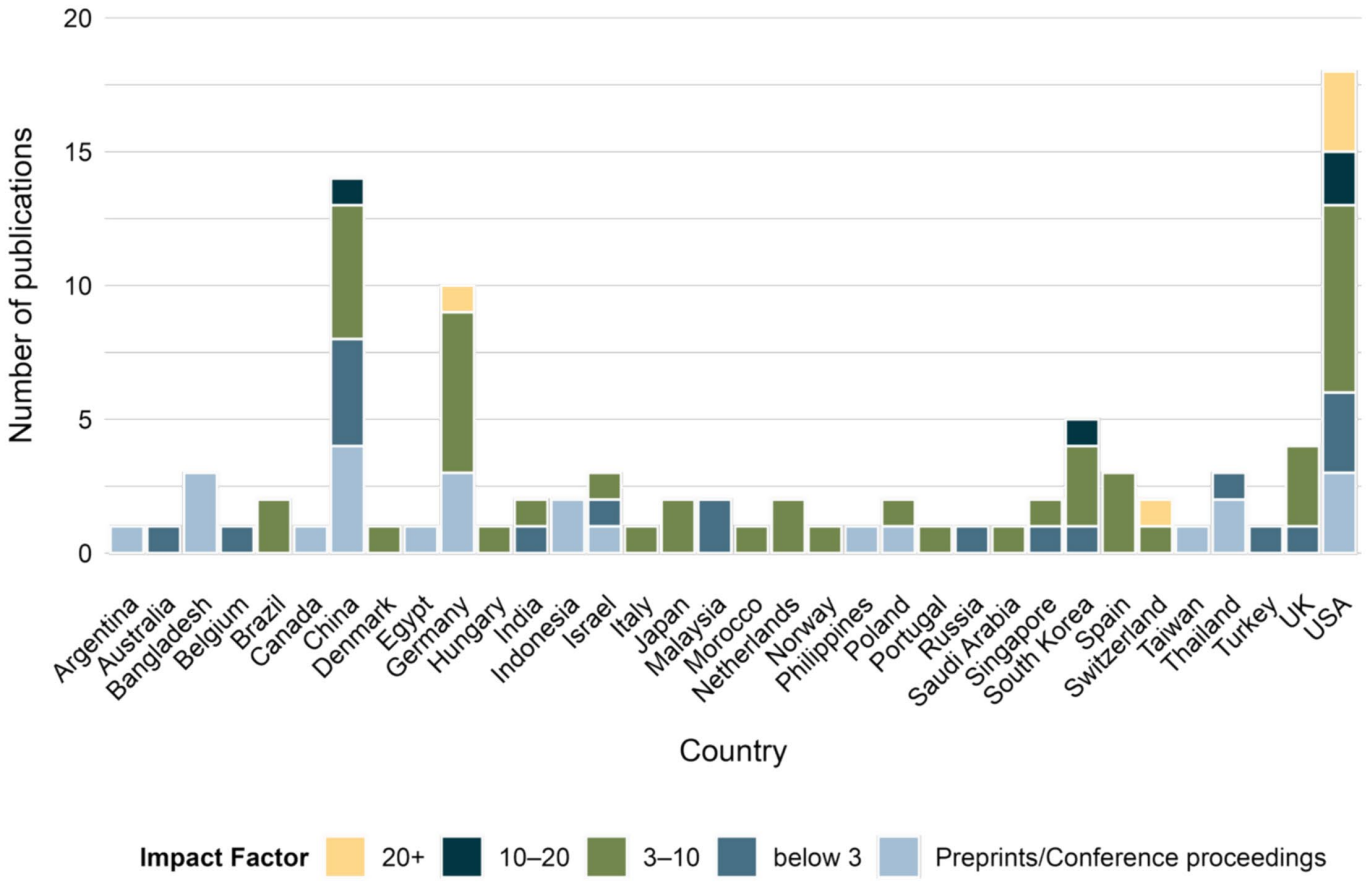
Number of articles published between 2015 and 2025 by country, grouped by journal’s impact factor.

Analysis of 97 publications, included in review, showed a growing interest in the field of AI-based research on behavior of invertebrate and larval model organisms. Starting from only 2 papers being published in 2015 (Girdhar et al., 2015; Stern et al., 2015), to 43 papers (34 in peer-reviewed journals (Girdhar et al., 2015; Stern et al., 2015; Shamur et al., 2016; Huang et al., 2016; Perni et al., 2018; Marques et al., 2018; Huang et al., 2018; Javer et al., 2018; Mathis et al., 2018; Aqil et al., 2018; Rudolf et al., 2019; Štih et al., 2019; Günel et al., 2019; Pereira et al., 2019; Bornhorst et al., 2019; Wu et al., 2019; Jeong et al., 2019; Jiang et al., 2019; Graving et al., 2019; Roosjen et al., 2020; Mearns et al., 2020; Layana Castro et al., 2020; Banerjee et al., 2020; Kukieattikool et al., 2020; Seong et al., 2020; Kasinathan et al., 2020; Karashchuk et al., 2021; Ong et al., 2021; García Garví et al., 2021; Gosztolai et al., 2021; Hebert et al., 2021; Ahmad et al., 2021; Bjerge et al., 2021; Michels et al., 2019) and 9 in preprints and conference proceedings (Bodenstein et al., 2016; Kesvarakul et al., 2017; Almarinez and Hernandez, 2019; Sharma et al., 2020; Suarez et al., 2021; Armalivia et al., 2021; Saeed et al., 2021; Gao et al., 2021; Bentzur et al., 2020) by 2021, and already 8 papers published in 2025 (Li et al., 2025; Yang et al., 2025; Arame et al., 2025; Zhang et al., 2025; Dang et al., 2025; Dolata et al., 2025; Zhan et al., 2025; McKenzie-Smith et al., 2025) (Figure 2; Supplementary Table S2). Notably, 43% of literature originated from the USA, China and Germany (Figure 3b), with 80% (Stern et al., 2015; Pereira et al., 2019; Karashchuk et al., 2021; Dang et al., 2025; McKenzie-Smith et al., 2025; Bates et al., 2022; Pereira et al., 2022; Ravan et al., 2023; Gore et al., 2023; Keleş et al., 2024; Gendelev et al., 2024; Melis et al., 2024), 60% (Huang et al., 2018; Li et al., 2025; Yang et al., 2025; Zhang et al., 2025; Zhan et al., 2025; Zhang et al., 2023) and 100% (Mathis et al., 2018; Štih et al., 2019; Bornhorst et al., 2019; Graving et al., 2019; Mearns et al., 2020; Michels et al., 2019; Bian et al., 2019), respectively being published in journals scored with impact factor 3 and above (Figure 4). This concentration of high-quality research output reflects the leading roles of these countries in both AI and life sciences fields (Al-Marzouqi and Arabi, 2024; Smith et al., 2019b). Countries in South America, Asia and Eastern Europe also undergo research in the field (Figure 3A, Figure 4), indicating global engagement possibly aided by widely accessible computational power for model development. Interestingly, most published research in the last decade appeared in journals with Impact Factor greater than 3 (74%) (Stern et al., 2015; Marques et al., 2018; Huang et al., 2018; Javer et al., 2018; Mathis et al., 2018; Rudolf et al., 2019; Štih et al., 2019; Günel et al., 2019; Pereira et al., 2019; Bornhorst et al., 2019; Wu et al., 2019; Jeong et al., 2019; Jiang et al., 2019; Graving et al., 2019; Roosjen et al., 2020; Mearns et al., 2020; Layana Castro et al., 2020; Seong et al., 2020; Kasinathan et al., 2020; Karashchuk et al., 2021; Ong et al., 2021; García Garví et al., 2021; Gosztolai et al., 2021; Hebert et al., 2021; Ahmad et al., 2021; Bjerge et al., 2021; Michels et al., 2019; Li et al., 2025; Yang et al., 2025; Arame et al., 2025; Zhang et al., 2025; Dang et al., 2025; Dolata et al., 2025; Zhan et al., 2025; McKenzie-Smith et al., 2025; Bates et al., 2022; Pereira et al., 2022; Ravan et al., 2023; Gore et al., 2023; Keleş et al., 2024; Gendelev et al., 2024; Melis et al., 2024; Zhang et al., 2023; Bian et al., 2019; Kang et al., 2024; Galimov and Yakimovich, 2022; Obasekore et al., 2023; Garcia-Garvi et al., 2023; Costa et al., 2023; Costa et al., 2022; Manduca et al., 2023; Zhang et al., 2024; Jang et al., 2024; Pham, 2023) (Figure 4). This places AI research on behavioral analysis of invertebrates and larval organisms as a significant, trending topic in the sub-field of science.

### Model organisms investigated

**Figure 5:**
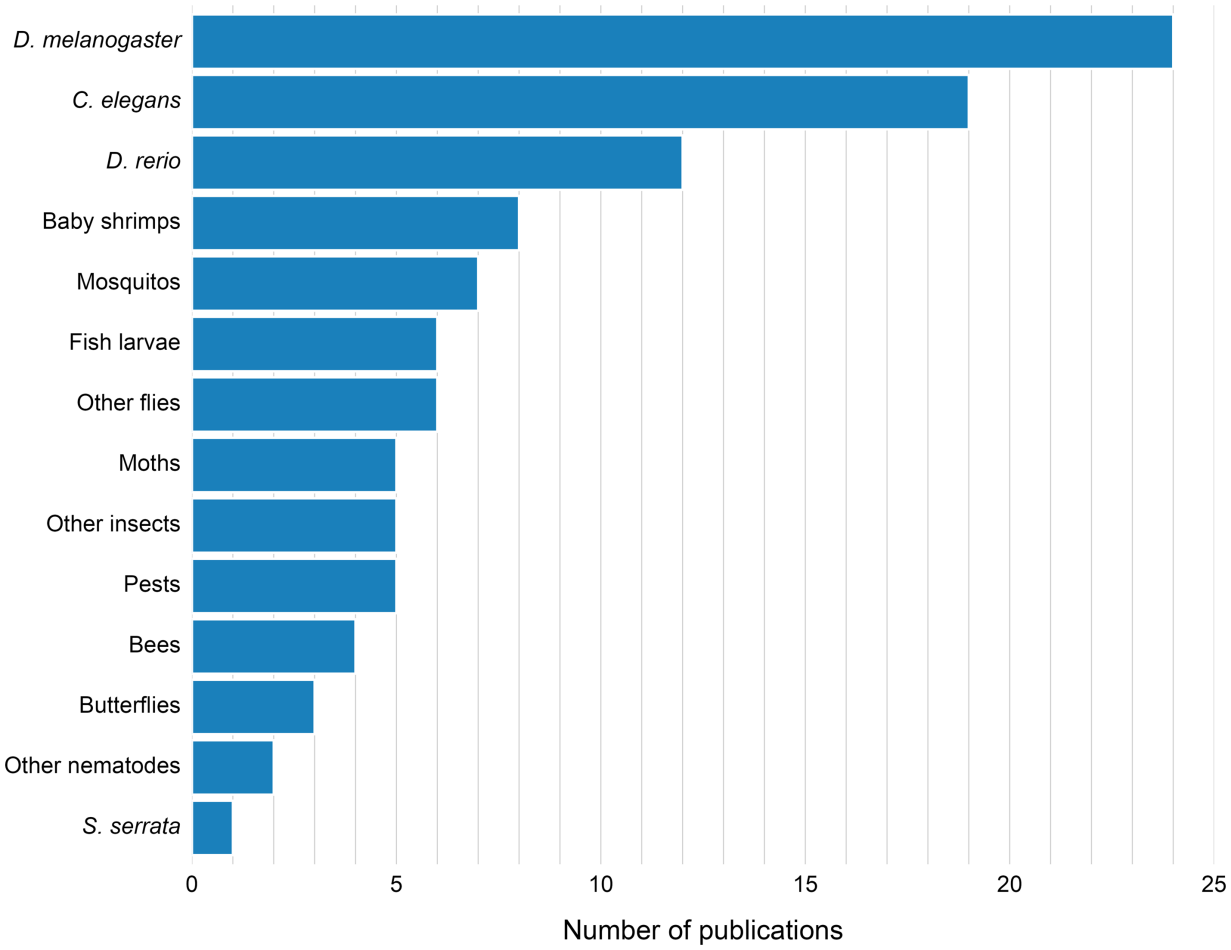
Model organisms investigated in analyzed research papers.

**Figure 6:**
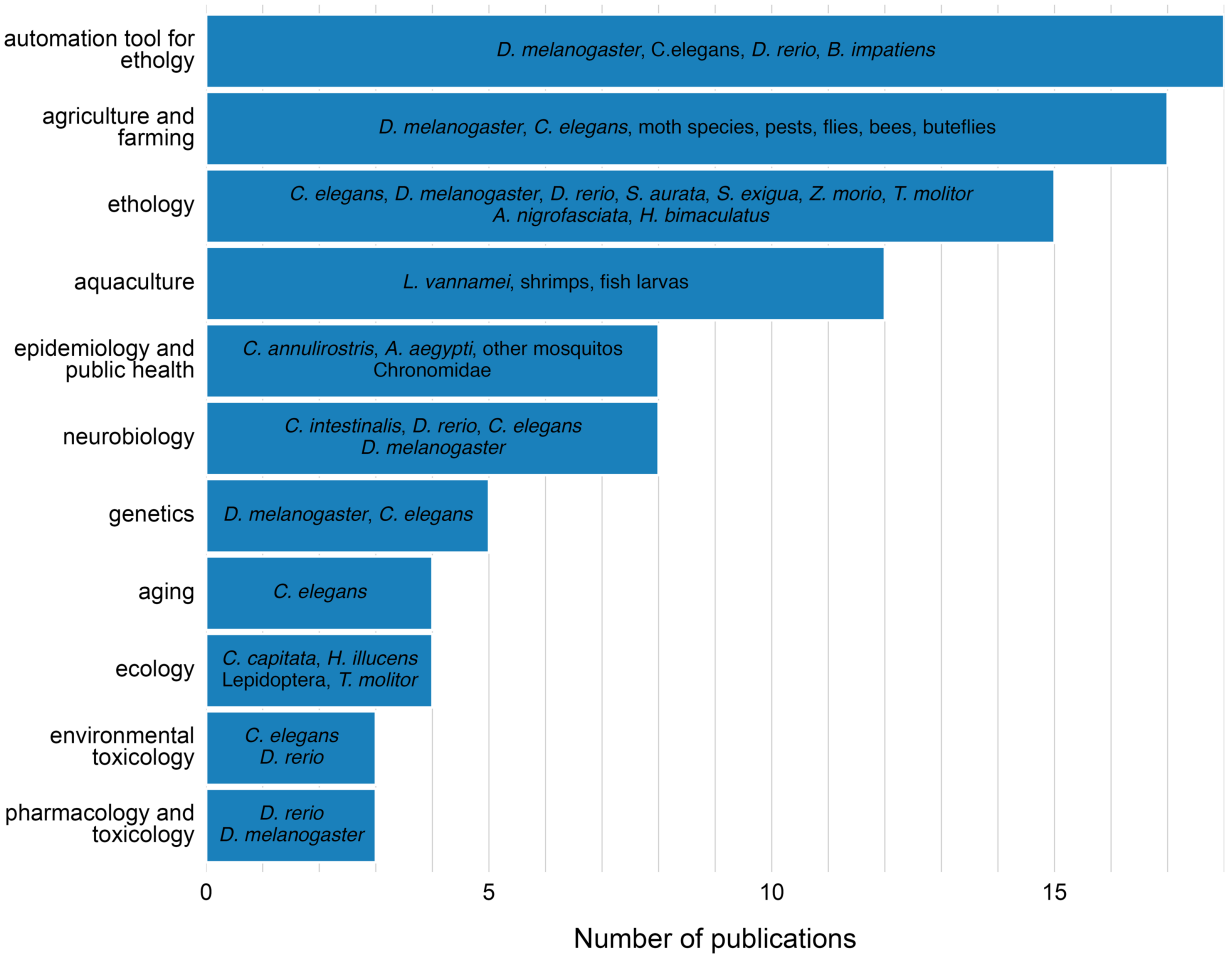
Investigated model organisms, according to the research field.

Over 50% of published research utilized widely recognized model organisms in biology, including *D. melanogaster* (22%) (Stern et al., 2015; Mathis et al., 2018; Günel et al., 2019; Pereira et al., 2019; Wu et al., 2019; Jiang et al., 2019; Graving et al., 2019; Banerjee et al., 2020; Seong et al., 2020; Karashchuk et al., 2021; Gosztolai et al., 2021; Michels et al., 2019; Bodenstein et al., 2016; Bentzur et al., 2020; Li et al., 2025; Yang et al., 2025; McKenzie-Smith et al., 2025; Pereira et al., 2022; Keleş et al., 2024; Melis et al., 2024; Bian et al., 2019; Genaev et al., 2022; Nguyen et al., 2022; Bigge et al., 2024), *C. elegans* (18%) (Huang et al., 2016; Perni et al., 2018; Javer et al., 2018; Bornhorst et al., 2019; Jeong et al., 2019; Layana Castro et al., 2020; García Garví et al., 2021; Hebert et al., 2021; Li et al., 2025; Zhan et al., 2025; Bates et al., 2022; Zhang et al., 2023; Kang et al., 2024; Galimov and Yakimovich, 2022; Garcia-Garvi et al., 2023; Pham, 2023; McClanahan et al., 2023; Banerjee et al., 2023; Weng et al., 2024) and *D. rerio*, in larval form (11%) (Girdhar et al., 2015; Marques et al., 2018; Štih et al., 2019; Pereira et al., 2019; Mearns et al., 2020; Li et al., 2025; Ravan et al., 2023; Gore et al., 2023; Gendelev et al., 2024; Green et al., 2023; Widrick et al., 2024; Si et al., 2022) (Figure 5; Supplementary Table S2). Within this group of organisms, research was conducted on larval forms in approximately 38% (Girdhar et al., 2015; Marques et al., 2018; Štih et al., 2019; Pereira et al., 2019; Mearns et al., 2020; Seong et al., 2020; Michels et al., 2019; Li et al., 2025; Bates et al., 2022; Ravan et al., 2023; Gore et al., 2023; Gendelev et al., 2024; Zhang et al., 2023; Bian et al., 2019; McClanahan et al., 2023; Green et al., 2023; Widrick et al., 2024; Si et al., 2022) (Supplementary Table S2). These organisms were chosen as model organisms across almost all identified research fields (Figure 6), including development of automation tools for ethology, genetics, ethology, agriculture and farming, neurobiology, aging, environmental toxicology, pharmacology and toxicology. Other significant groups of model organisms investigated were shrimps (incl. *Litopenaeus vannamei*) (Kesvarakul et al., 2017; Armalivia et al., 2021; Zhang et al., 2025; Dang et al., 2025; Long et al., 2023; Zhang et al., 2024; Zhou et al., 2023; Tiyapunjanit et al., 2023) and mosquitos (incl. *Culex annulirostris, Aedes aegypti*) (Aqil et al., 2018; Kukieattikool et al., 2020; Saeed et al., 2021; Surya et al., 2022; Abedin et al., 2023; Jahangir et al., 2023; Javed et al., 2024), mainly investigated in aquaculture and epidemiology, respectively (Figure 6; Supplementary Table S2). Moreover, various pests, moths, fish larva and insect species found applications in agricultural and farming (Huang et al., 2018; Roosjen et al., 2020; Kasinathan et al., 2020; Ong et al., 2021; Ahmad et al., 2021; Suarez et al., 2021; Gao et al., 2021; Arame et al., 2025; Obasekore et al., 2023; Lee and Gao, 2023; Fawzy et al., 2024; Majewski et al., 2024; Gunes and Gungormus, 2022; Nwoko et al., 2023; Liu et al., 2024; Ahmad et al., 2024), aquacultural (Almarinez and Hernandez, 2019; Costa et al., 2023; Costa et al., 2022; Zhang et al., 2024), ethological (Shamur et al., 2016; Deng et al., 2024; Rujito et al., 2023) and ecological studies (Figure 6).

### Overview of models used in behavioral research

**Figure 7:**
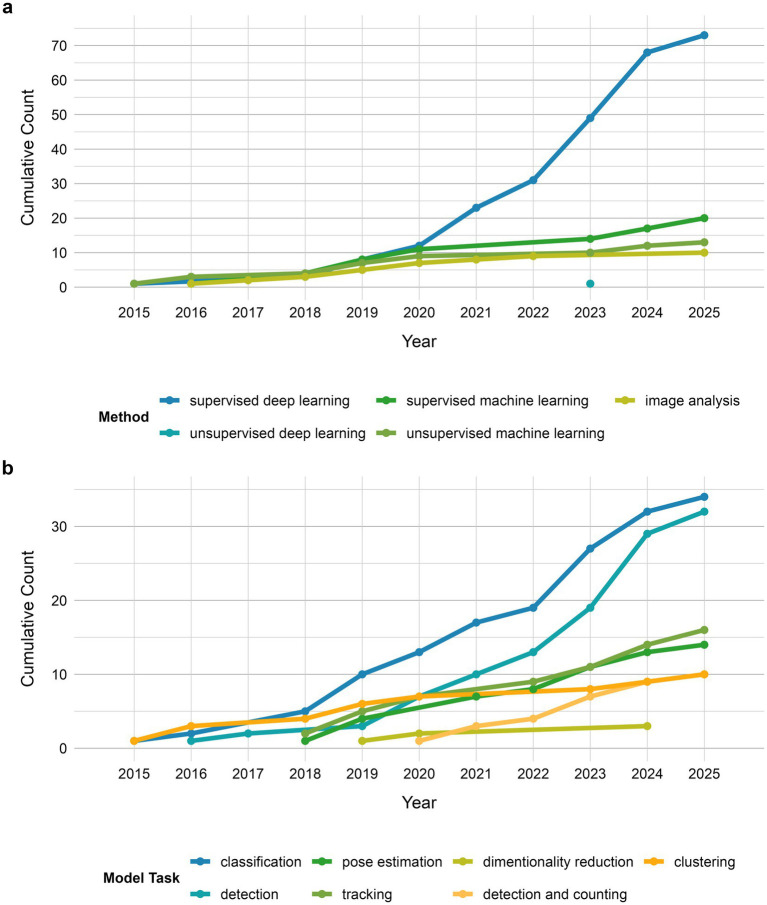
Overview of AI-based approaches in behavioral analysis of invertebrate and larval model organisms. Grouped by method **(b)** and model task **(b)**.

Supervised deep learning was the dominant method used in the field of AI-based behavioral analysis of invertebrate and larval organisms, followed by supervised machine learning and unsupervised machine learning (Figure 7a). The less frequently chosen model group was image analysis, defined as semi-automatic approach consisting of the pipeline of code that processes input data without the element of learning (Figure 7a). Some of the examples of algorithms used in this group of models include the Adaptive Shape Model (Bian et al., 2019), or adaptive thresholding incorporated into analysis pipeline (Perni et al., 2018). Statistical analysis was only used three times (e.g., Particle filtering or Reversible Jump Markov Chain Monte Carlo algorithm) (Seong et al., 2020; Michels et al., 2019; Yin et al., 2024). The method appearing only once within analyzed literature was unsupervised deep learning. A deep autoencoder was used to classify normal/abnormal zebrafish larvae behavior (Green et al., 2023). Until 2020 the development of AI-based methods was balanced between the four main groups: sDL, sML, uML and image analysis algorithms, appearing in 12 (29%) (Stern et al., 2015; Mathis et al., 2018; Aqil et al., 2018; Günel et al., 2019; Pereira et al., 2019; Jeong et al., 2019; Jiang et al., 2019; Graving et al., 2019; Roosjen et al., 2020; Seong et al., 2020; Kasinathan et al., 2020; Sharma et al., 2020), 11 (27%) (Shamur et al., 2016; Huang et al., 2018; Javer et al., 2018; Rudolf et al., 2019; Bornhorst et al., 2019; Wu et al., 2019; Banerjee et al., 2020; Kukieattikool et al., 2020; Almarinez and Hernandez, 2019; Bentzur et al., 2020), 9 (22%) (Girdhar et al., 2015; Huang et al., 2016; Marques et al., 2018; Rudolf et al., 2019; Jeong et al., 2019; Mearns et al., 2020; Bodenstein et al., 2016) and 7 (17%) (Perni et al., 2018; Štih et al., 2019; Layana Castro et al., 2020; Bodenstein et al., 2016; Kesvarakul et al., 2017; Almarinez and Hernandez, 2019; Bentzur et al., 2020) papers, respectively (Figure 7a). After 2020, however, the number of papers focusing on developing sDL algorithms skyrocketed to the total of 73 (62%) (Stern et al., 2015; Mathis et al., 2018; Aqil et al., 2018; Günel et al., 2019; Pereira et al., 2019; Jeong et al., 2019; Jiang et al., 2019; Graving et al., 2019; Roosjen et al., 2020; Seong et al., 2020; Kasinathan et al., 2020; Karashchuk et al., 2021; Ong et al., 2021; García Garví et al., 2021; Gosztolai et al., 2021; Hebert et al., 2021; Ahmad et al., 2021; Bjerge et al., 2021; Sharma et al., 2020; Suarez et al., 2021; Armalivia et al., 2021; Saeed et al., 2021; Gao et al., 2021; Li et al., 2025; Yang et al., 2025; Zhang et al., 2025; Zhan et al., 2025; McKenzie-Smith et al., 2025; Bates et al., 2022; Pereira et al., 2022; Ravan et al., 2023; Gore et al., 2023; Keleş et al., 2024; Gendelev et al., 2024; Melis et al., 2024; Zhang et al., 2023; Kang et al., 2024; Galimov and Yakimovich, 2022; Obasekore et al., 2023; Garcia-Garvi et al., 2023; Costa et al., 2023; Costa et al., 2022; Manduca et al., 2023; Zhang et al., 2024; Jang et al., 2024; Pham, 2023; Genaev et al., 2022; Bigge et al., 2024; McClanahan et al., 2023; Banerjee et al., 2023; Weng et al., 2024; Widrick et al., 2024; Si et al., 2022; Long et al., 2023; Zhang et al., 2024; Zhou et al., 2023; Tiyapunjanit et al., 2023; Surya et al., 2022; Abedin et al., 2023; Jahangir et al., 2023; Javed et al., 2024; Lee and Gao, 2023; Fawzy et al., 2024; Majewski et al., 2024; Gunes and Gungormus, 2022; Nwoko et al., 2023; Liu et al., 2024; Ahmad et al., 2024; Deng et al., 2024; Rujito et al., 2023; Yin et al., 2024) by 2025, while supervised and unsupervised ML approaches grew in popularity at a steady rate, adding up to 20 (17%) (Shamur et al., 2016; Huang et al., 2018; Javer et al., 2018; Rudolf et al., 2019; Bornhorst et al., 2019; Wu et al., 2019; Banerjee et al., 2020; Kukieattikool et al., 2020; Almarinez and Hernandez, 2019; Bentzur et al., 2020; Arame et al., 2025; Dang et al., 2025; McKenzie-Smith et al., 2025; Keleş et al., 2024; Gendelev et al., 2024; Manduca et al., 2023; Nguyen et al., 2022; McClanahan et al., 2023; Widrick et al., 2024) and 13 (11%) (Girdhar et al., 2015; Huang et al., 2016; Marques et al., 2018; Rudolf et al., 2019; Jeong et al., 2019; Mearns et al., 2020; Bodenstein et al., 2016; McKenzie-Smith et al., 2025; Gore et al., 2023; Keleş et al., 2024; Gendelev et al., 2024), respectively (Figure 7A). The drastic increase in development of sDL approaches can be attributed, to some degree, to the development of CNN-based markerless pose estimation tools called to such as DeepLabCut (Mathis et al., 2018) and SLEAP (Pereira et al., 2022), which became widely used tools in behavioral analysis and precursors for other pose estimation algorithms (e.g., Z-LAP tracker Gore et al., 2023).

Investigating types of tasks models performed, the dominant ones are classification (Stern et al., 2015; Shamur et al., 2016; Huang et al., 2018; Javer et al., 2018; Aqil et al., 2018; Rudolf et al., 2019; Bornhorst et al., 2019; Jeong et al., 2019; Jiang et al., 2019; Kukieattikool et al., 2020; Kasinathan et al., 2020; García Garví et al., 2021; Bjerge et al., 2021; Almarinez and Hernandez, 2019; Suarez et al., 2021; Saeed et al., 2021; Bentzur et al., 2020; Arame et al., 2025; McKenzie-Smith et al., 2025; Keleş et al., 2024; Gendelev et al., 2024; Kang et al., 2024; Galimov and Yakimovich, 2022; Manduca et al., 2023; Pham, 2023; Nguyen et al., 2022; McClanahan et al., 2023; Green et al., 2023; Widrick et al., 2024; Tiyapunjanit et al., 2023; Surya et al., 2022; Abedin et al., 2023; Rujito et al., 2023) and detection (Roosjen et al., 2020; Layana Castro et al., 2020; Seong et al., 2020; Bodenstein et al., 2016; Kesvarakul et al., 2017; Almarinez and Hernandez, 2019; Armalivia et al., 2021; Gao et al., 2021; Zhang et al., 2025; Dolata et al., 2025; Zhan et al., 2025; Zhang et al., 2023; Bian et al., 2019; Galimov and Yakimovich, 2022; Obasekore et al., 2023; Costa et al., 2023; Zhang et al., 2024; Jang et al., 2024; Genaev et al., 2022; Bigge et al., 2024; Weng et al., 2024; Zhang et al., 2024; Jahangir et al., 2023; Lee and Gao, 2023; Fawzy et al., 2024; Majewski et al., 2024; Gunes and Gungormus, 2022; Nwoko et al., 2023; Liu et al., 2024; Deng et al., 2024; Yin et al., 2024) (34 and 32 uses, respectively), followed by tracking (Perni et al., 2018; Javer et al., 2018; Štih et al., 2019; Wu et al., 2019; Banerjee et al., 2020; Michels et al., 2019; Bentzur et al., 2020; Yang et al., 2025; McKenzie-Smith et al., 2025; Bates et al., 2022; Melis et al., 2024; McClanahan et al., 2023; Banerjee et al., 2023; Si et al., 2022; Deng et al., 2024; Yin et al., 2024) and pose estimation (Mathis et al., 2018; Günel et al., 2019; Pereira et al., 2019; Graving et al., 2019; Karashchuk et al., 2021; Gosztolai et al., 2021; Hebert et al., 2021; Li et al., 2025; Pereira et al., 2022; Ravan et al., 2023; Gore et al., 2023; Keleş et al., 2024; Manduca et al., 2023; Widrick et al., 2024) (16 and 14, respectively), clustering (Girdhar et al., 2015; Huang et al., 2016; Marques et al., 2018; Rudolf et al., 2019; Jeong et al., 2019; Mearns et al., 2020; Bodenstein et al., 2016; McKenzie-Smith et al., 2025; Gore et al., 2023; Gendelev et al., 2024) and detection and counting (Ong et al., 2021; Ahmad et al., 2021; Sharma et al., 2020; Dang et al., 2025; Garcia-Garvi et al., 2023; Costa et al., 2022; Long et al., 2023; Zhou et al., 2023; Javed et al., 2024; Ahmad et al., 2024) (10 uses, each), and less common dimensionality reduction techniques (Jeong et al., 2019; Mearns et al., 2020; Keleş et al., 2024) (3 uses) (Figure 7B).

**Figure 8:**
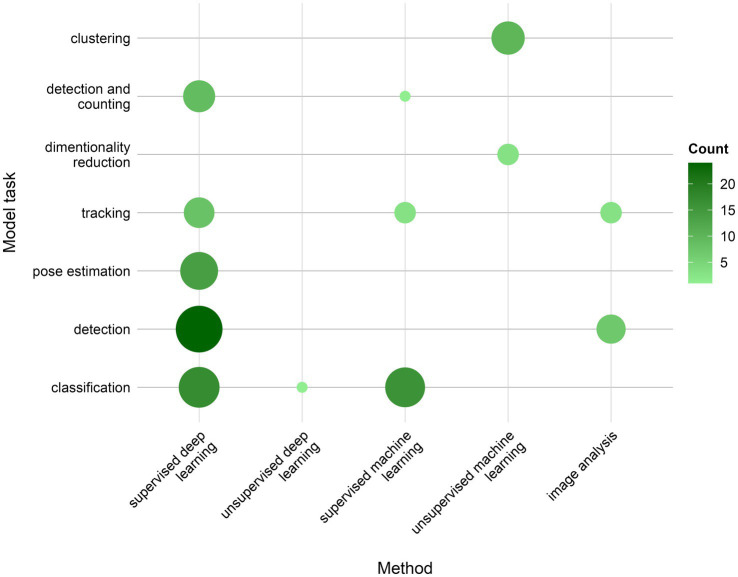
Distribution of AI models’ tasks used in different types of methods.

Looking at the models’ profiles, grouped by method and type of task performed (Figure 8), sDL was used in almost all classes of tasks, as an exclusive approach for pose estimation as well as the dominant approach in detection and detection and counting tasks. sDL and sML methods were used in similar relative frequency in classification tasks. Tracking tasks used almost all methods, while clustering, and dimensionality reduction tasks, as an inherently separate group of tasks, used unsupervised learning approaches.

Diving deeper into the specific algorithms, within a subgroup of sDL approaches, the most common algorithms used were based on CNN architecture, and included YOLO (You Only Look Once) algorithms (Armalivia et al., 2021; Gao et al., 2021; Zhang et al., 2023; Jang et al., 2024; Genaev et al., 2022; Bigge et al., 2024; Banerjee et al., 2023; Weng et al., 2024; Si et al., 2022; Majewski et al., 2024; Nwoko et al., 2023; Liu et al., 2024; Deng et al., 2024) (13 uses), Faster R-CNN (Ahmad et al., 2021; Bates et al., 2022; Obasekore et al., 2023; Garcia-Garvi et al., 2023; Costa et al., 2022; Gunes and Gungormus, 2022; Ahmad et al., 2024) (7 uses), markerless pose estimation tool DeepLabCut (Mathis et al., 2018; Karashchuk et al., 2021; Keleş et al., 2024; Manduca et al., 2023; Widrick et al., 2024) (5 uses), along with other pose estimation tools that either were directly based on DeepLabCut [Z-Lap Tracker (Gore et al., 2023)] or employed a similar algorithm (LEAP (Pereira et al., 2019), SLEEP (Pereira et al., 2022) and WormPose (Hebert et al., 2021). A less common, yet increasingly developed sDL methods that appeared in published research were transformers and RNN architectures. While transformers (RT-DETR, VITPose and KAN Transformer) were used in variety of tasks (detection (Zhang et al., 2024; Fawzy et al., 2024), pose estimation (Li et al., 2025) and tracking (Yang et al., 2025, respectively), RNN models (LSTM) were used twice for classification of *C. elegans* swimming patterns (Kang et al., 2024; Pham, 2023) and once for predicting larval movement trajectory 1.5 s into the future (Deng et al., 2024).

Taking a closer look at the supervised machine learning models, the three most commonly used algorithms included Support Vector Machines (SVM), Random Forest (RF) and K-nearest neighbors (KNN). These models are being used as a stand-alone classification algorithm or folowed by simple image analysis (4 (Shamur et al., 2016; Huang et al., 2018; Bornhorst et al., 2019; Kukieattikool et al., 2020), 2 (Arame et al., 2025; Nguyen et al., 2022) and 1 use (Almarinez and Hernandez, 2019) use, respectively), in conjunction with pose estimation tool to classify poses into investigated phenotypes (2 (McKenzie-Smith et al., 2025; Widrick et al., 2024), 3 (Manduca et al., 2023; Widrick et al., 2024) and 1 (Manduca et al., 2023) use, respectively) and less commonly as an initial classifier to reduce dimensionality for deep learning (RF and KNN followed by Siamese Neural Networks Twin-NN an Twin-DN (Gendelev et al., 2024), or following unsupervised clustering into behaviorally distinct groups [agglomerative clustering algorithm followed by KNN (Rudolf et al., 2019)].

The unsupervised machine learning was most commonly applied in clustering algorithms (Girdhar et al., 2015; Huang et al., 2016; Marques et al., 2018; Rudolf et al., 2019; Jeong et al., 2019; Mearns et al., 2020; Bodenstein et al., 2016; Gore et al., 2023) (e.g., k-means clustering, MotionMapper, n = 8) and less commonly in dimensionality reduction (Mearns et al., 2020; Keleş et al., 2024; Gendelev et al., 2024) (e.g., UMAP, SOM, n = 3). The clustering method was used to group together animal poses (McKenzie-Smith et al., 2025; Gore et al., 2023; Keleş et al., 2024), as a stand-alone tool to group behavioral patterns such as swimming or movement patterns (Girdhar et al., 2015; Huang et al., 2016; Marques et al., 2018), and in conjunction with other approaches to cluster behavior as a preparation step for further ML or DL analysis (Rudolf et al., 2019; Jeong et al., 2019).

Finally, image analysis approach, was documented to be used until 2021 in 8 papers (Figure 7), as a stand-alone tool for detection or tracking (Perni et al., 2018; Štih et al., 2019; Layana Castro et al., 2020; Kesvarakul et al., 2017; Bian et al., 2019), as a preprocessing step for classical supervised learning (Almarinez and Hernandez, 2019; Bentzur et al., 2020) and as a detection technique for clustered animals (Rudolf et al., 2019). It is possible that with the development of sDL approaches and higher complexity of behavioral research, image analysis was less frequently reported in published methodology or even neglected in favor of presenting end-to-end sDL pipelines.

Of note, several sDL models (CNN-based models incl. YOLO and Faster R-CNN) had been pretrained on COCO or ImageNet datasets for detection and classification tasks (Dolata et al., 2025; Zhan et al., 2025; Bates et al., 2022; Keleş et al., 2024; Kang et al., 2024; Obasekore et al., 2023; Jang et al., 2024; Genaev et al., 2022; Bigge et al., 2024; Banerjee et al., 2023; Surya et al., 2022; Abedin et al., 2023; Gunes and Gungormus, 2022). This procedure accelerated training, allowing for a smaller number of input data required to achieve high accuracy results. However, it can be seen from model’s task profile, pretraining had only been advantageous for relatively simple detection tasks. On the other hand, transfer learning had been used in a wider, and more computationally demanding group of tasks, such as CNN-based algorithms for classification and pose estimation (Mathis et al., 2018; Aqil et al., 2018; Karashchuk et al., 2021; Ong et al., 2021; Tiyapunjanit et al., 2023; Yin et al., 2024). Finally, 2 papers utilized active learning for pose estimation tasks (Günel et al., 2019; Pereira et al., 2019) allowing for further training in real-time without user intervention. One of the examples of classification into abnormal and normal swimming patterns used semi-supervised approach (Green et al., 2023), meaning the model was trained on control data only and was able to classify abnormal behavior as a distinct pattern from learnt control dataset.

### Overview of input data

Most experimental data for model training came from in-lab experiments with only 14% of papers training models on public datasets (Javer et al., 2018; Jiang et al., 2019; Kasinathan et al., 2020; Michels et al., 2019; Zhan et al., 2025; Bian et al., 2019; Galimov and Yakimovich, 2022; Pham, 2023; Banerjee et al., 2023; Long et al., 2023; Surya et al., 2022; Jahangir et al., 2023; Fawzy et al., 2024; Rujito et al., 2023). The most popular data acquisition technique was image (RGB, monochrome or greyscale) or video capture using a single camera (Supplementary Table S3). Less frequently researchers used:

- Multiple synchronized camera set-up (sDL models for pose estimation (Günel et al., 2019; Karashchuk et al., 2021; Gosztolai et al., 2021), tracking (Melis et al., 2024; Yin et al., 2024) and classification (Stern et al., 2015) tasks)
- Specialized cameras such as hyperspectral (Huang et al., 2018; Arame et al., 2025; Jang et al., 2024) or CCD cameras (Jeong et al., 2019; Seong et al., 2020; Kang et al., 2024)
- Cartesian multi-view robot (sDL model for detection and counting (Garcia-Garvi et al., 2023)
- Specialized laboratory incubators with tracking systems (e.g., ZebraBox (Green et al., 2023), FlyBowl (Bentzur et al., 2020), WormTracker (Huang et al., 2016), Ethovision XT (Nguyen et al., 2022; Green et al., 2023).

Interestingly, 11 papers used high-speed cameras to capture animal behavior (Girdhar et al., 2015; Shamur et al., 2016; Marques et al., 2018; Štih et al., 2019; Wu et al., 2019; Mearns et al., 2020; Banerjee et al., 2020; Almarinez and Hernandez, 2019; Yang et al., 2025; McKenzie-Smith et al., 2025; Widrick et al., 2024) while 3 used IR-sensitive cameras (Marques et al., 2018; Rudolf et al., 2019; Štih et al., 2019). In 10 experiments data was acquired via a smartphone camera (Bornhorst et al., 2019; Kukieattikool et al., 2020; Armalivia et al., 2021; Costa et al., 2023; Costa et al., 2022; Genaev et al., 2022; Si et al., 2022; Zhou et al., 2023; Tiyapunjanit et al., 2023; Liu et al., 2024). Finally, 5 papers generated synthetic data (Hebert et al., 2021; Sharma et al., 2020; Dolata et al., 2025; Ravan et al., 2023; Garcia-Garvi et al., 2023), on top of in-lab acquired material.

Unfortunately, the level of detail on source of input data was not reported cohesively across the literature. While some papers reported detailed specifications on input data (e.g., resolution, recording speed in a form of frames per second (fps), shutter speed) or camera used (e.g., model, filters, lenses), others did not report any details, even as basic as camera model (Supplementary Table S3).

**Figure 9:**
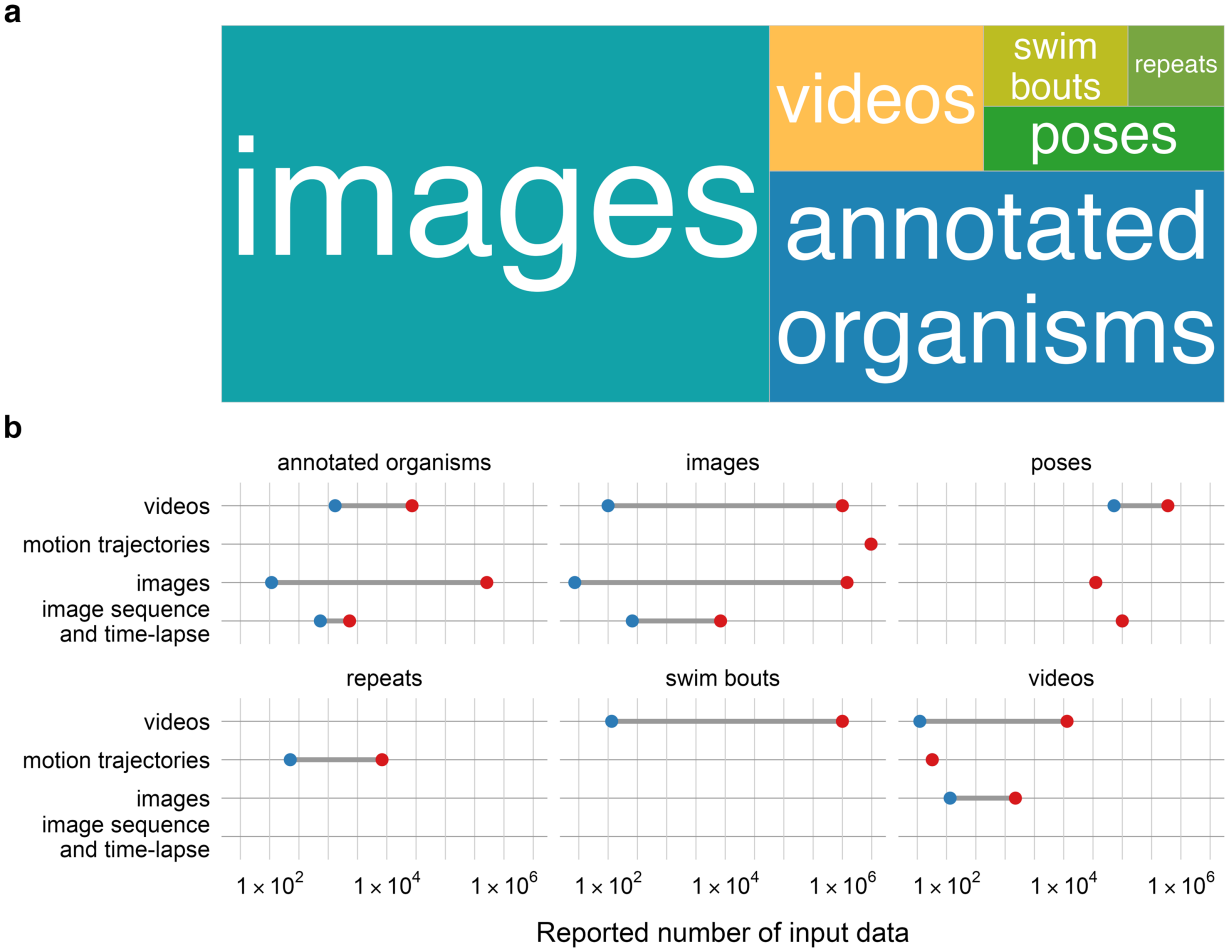
Overview of input data reported in analyzed studies. Type of input data reported, size of boxes relative to the total number of publications reporting stated type **(a)**, total number of input data used for model training according to the type of input data **(b)**. The blue and red dots mark minimum and maximum number of input data on the range scale.

This heterogeneity in reported data is also visible in the format of reporting input data used to train the model. As seen on Figure 9a authors reported quantity of input data in the units of images (54%) (Perni et al., 2018; Huang et al., 2018; Mathis et al., 2018; Aqil et al., 2018; Rudolf et al., 2019; Štih et al., 2019; Pereira et al., 2019; Wu et al., 2019; Jeong et al., 2019; Jiang et al., 2019; Graving et al., 2019; Banerjee et al., 2020; Kasinathan et al., 2020; Ong et al., 2021; Gosztolai et al., 2021; Hebert et al., 2021; Ahmad et al., 2021; Bodenstein et al., 2016; Kesvarakul et al., 2017; Almarinez and Hernandez, 2019; Armalivia et al., 2021; Saeed et al., 2021; Gao et al., 2021; Li et al., 2025; Yang et al., 2025; Arame et al., 2025; Dolata et al., 2025; Gore et al., 2023; Keleş et al., 2024; Bian et al., 2019; Kang et al., 2024; Obasekore et al., 2023; Garcia-Garvi et al., 2023; Zhang et al., 2024; Jang et al., 2024; Pham, 2023; Genaev et al., 2022; Banerjee et al., 2023; Weng et al., 2024; Green et al., 2023; Si et al., 2022; Zhou et al., 2023; Tiyapunjanit et al., 2023; Abedin et al., 2023; Jahangir et al., 2023; Lee and Gao, 2023; Fawzy et al., 2024; Gunes and Gungormus, 2022; Liu et al., 2024; Ahmad et al., 2024; Rujito et al., 2023; Yin et al., 2024), annotated organisms (27%) (Stern et al., 2015; Günel et al., 2019; Bornhorst et al., 2019; Seong et al., 2020; Karashchuk et al., 2021; Bjerge et al., 2021; Suarez et al., 2021; Zhang et al., 2025; Dang et al., 2025; Zhan et al., 2025; McKenzie-Smith et al., 2025; Bates et al., 2022; Pereira et al., 2022; Zhang et al., 2023; Galimov and Yakimovich, 2022; Costa et al., 2023; Costa et al., 2022; Manduca et al., 2023; Bigge et al., 2024; McClanahan et al., 2023; Long et al., 2023; Zhang et al., 2024; Surya et al., 2022; Majewski et al., 2024; Nwoko et al., 2023; Deng et al., 2024), videos (8%) (Shamur et al., 2016; Huang et al., 2016; Javer et al., 2018; Kukieattikool et al., 2020; García Garví et al., 2021; Ravan et al., 2023; Widrick et al., 2024; Javed et al., 2024), poses and swim bouts (specific type of pose especially for *D. rerio* larva) (7%) (Girdhar et al., 2015; Marques et al., 2018; Mearns et al., 2020; Layana Castro et al., 2020; Michels et al., 2019; Bentzur et al., 2020; Melis et al., 2024) and least commonly motion trajectories (4%) (Huang et al., 2016; Gendelev et al., 2024; Nguyen et al., 2022; Green et al., 2023). Moreover, as seen in Figure 9b, the actual number of input data ranges from 100-1,000,000 in all input data categories, from less than 100 input data (in a form of videos) to over 1,000,000 input data (in a form of images).

### Methods for behavior characterization and quantification

The portion of literature focused on analyzing behavior of model organisms (Girdhar et al., 2015; Shamur et al., 2016; Huang et al., 2016; Perni et al., 2018; Marques et al., 2018; Javer et al., 2018; Mathis et al., 2018; Rudolf et al., 2019; Štih et al., 2019; Günel et al., 2019; Pereira et al., 2019; Wu et al., 2019; Jeong et al., 2019; Jiang et al., 2019; Mearns et al., 2020; Banerjee et al., 2020; Kukieattikool et al., 2020; Kasinathan et al., 2020; Karashchuk et al., 2021; García Garví et al., 2021; Gosztolai et al., 2021; Hebert et al., 2021; Michels et al., 2019; Bentzur et al., 2020; Li et al., 2025; Yang et al., 2025; McKenzie-Smith et al., 2025; Pereira et al., 2022; Ravan et al., 2023; Gore et al., 2023; Keleş et al., 2024; Gendelev et al., 2024; Melis et al., 2024; Bian et al., 2019; Kang et al., 2024; Galimov and Yakimovich, 2022; Manduca et al., 2023; Nguyen et al., 2022; McClanahan et al., 2023; Green et al., 2023; Widrick et al., 2024; Surya et al., 2022; Rujito et al., 2023) (43 papers, Supplementary Table S3), rather than their detection, employed several main strategies for extracting behavior characteristics. The most common strategies included:

- Training algorithm to recognize defined model organisms’ shapes (Kasinathan et al., 2020; Bian et al., 2019; McClanahan et al., 2023; Surya et al., 2022), feeding behaviors (Shamur et al., 2016), body parts (Galimov and Yakimovich, 2022; Surya et al., 2022) or social interactions (Jiang et al., 2019),
- Tracking animals’ movement trajectories, shapes or poses to calculate kinematic movement parameters such as distance traveled, speed or stop duration (Perni et al., 2018; Štih et al., 2019; Banerjee et al., 2020; Bentzur et al., 2020; Li et al., 2025; Gendelev et al., 2024; Manduca et al., 2023; Green et al., 2023).
- Extract poses of animals for tracking experiments or to characterize behavior (Mathis et al., 2018; Pereira et al., 2019; Karashchuk et al., 2021; Hebert et al., 2021; McKenzie-Smith et al., 2025; Pereira et al., 2022; Ravan et al., 2023; Melis et al., 2024),
- Extracting animal poses to classify or cluster behavior into groups (treated /naïve, wild-type/mutant, long-lived/short-lived) (Rudolf et al., 2019; Gendelev et al., 2024; Galimov and Yakimovich, 2022; Nguyen et al., 2022; Widrick et al., 2024),
- Tracking animals’ movement trajectories to cluster or classify movement patterns (Girdhar et al., 2015; Marques et al., 2018; Jeong et al., 2019; Gore et al., 2023; Keleş et al., 2024).

**Figure 10:**
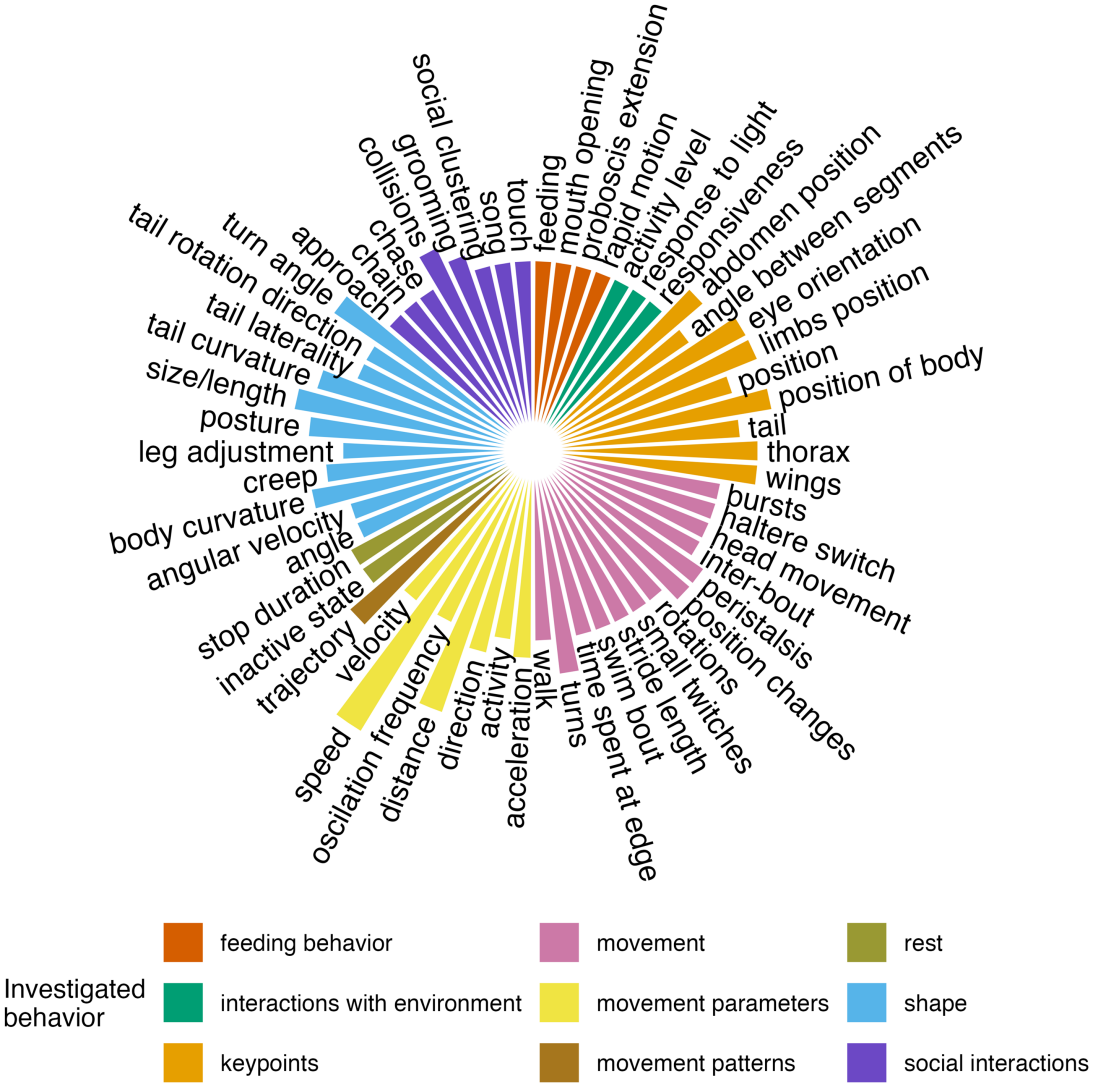
Overview of reported behavioral characteristics investigated during the analysis. The behavioral characteristics are color-grouped, and the size of bars represents relative frequency of studying behavioral characteristics.

These strategies either formulated the main core of the study characterizing organism’s behavior, discovered new movement patterns or were further used to assess behavior of model organisms under different physical and chemical conditions, inferring on animal lifespan or genotype. The behavioral characteristics investigated in analyzed papers are illustrated on Figure 10.

The most widely used behavioral characteristics fall into the group of movement, movement parameters and organisms’ shapes (Figure 10). Features such as total distance, direction of travel, or speed were readily calculated from tracking or pose estimation algorithms (Javer et al., 2018; Banerjee et al., 2020; Michels et al., 2019; Bentzur et al., 2020; Li et al., 2025; Gore et al., 2023; Widrick et al., 2024). On the other hand, movement features and shapes were most identified by the supervised machine learning and deep learning algorithms, which learnt from manually labeled data (Shamur et al., 2016; Jiang et al., 2019; Kukieattikool et al., 2020; Kasinathan et al., 2020; García Garví et al., 2021; Gendelev et al., 2024; Galimov and Yakimovich, 2022; Nguyen et al., 2022; Green et al., 2023; Surya et al., 2022). Another group of behavioral characteristics widely used were keypoints (Mathis et al., 2018; Günel et al., 2019; Pereira et al., 2019; Karashchuk et al., 2021; Hebert et al., 2021; Li et al., 2025; Pereira et al., 2022; Ravan et al., 2023; Gore et al., 2023; Keleş et al., 2024; Manduca et al., 2023), that found applications in pose estimation models (Figure 10). Depending on the organism investigated, different sets of features are most informative. For instance, worms’ behavior was most commonly quantified in the form of shapes (such as coils, curvature, angle, length) (Huang et al., 2016; Perni et al., 2018; Javer et al., 2018; Jeong et al., 2019; García Garví et al., 2021; Hebert et al., 2021; Li et al., 2025; Kang et al., 2024; Galimov and Yakimovich, 2022; McClanahan et al., 2023), whereas social behavior was almost exclusively investigated in flies and flying insects (Shamur et al., 2016; Jiang et al., 2019). Feeding strikes and swimming patterns were the most frequent in studies on larval form of zebrafish (Marques et al., 2018; Štih et al., 2019; Widrick et al., 2024). The overwhelming majority of behavioral analysis was investigated on *D. rerio* (Girdhar et al., 2015; Marques et al., 2018; Štih et al., 2019; Mearns et al., 2020; Ravan et al., 2023; Gore et al., 2023; Gendelev et al., 2024; Green et al., 2023; Widrick et al., 2024), *D. melanogaster* (Mathis et al., 2018; Günel et al., 2019; Pereira et al., 2019; Wu et al., 2019; Jiang et al., 2019; Banerjee et al., 2020; Karashchuk et al., 2021; Gosztolai et al., 2021; Michels et al., 2019; Bentzur et al., 2020; Li et al., 2025; Yang et al., 2025; McKenzie-Smith et al., 2025; Pereira et al., 2022; Keleş et al., 2024; Melis et al., 2024; Bian et al., 2019; Nguyen et al., 2022) and *C. elegans* (Huang et al., 2016; Perni et al., 2018; Javer et al., 2018; Jeong et al., 2019; García Garví et al., 2021; Hebert et al., 2021; Li et al., 2025; Kang et al., 2024; Galimov and Yakimovich, 2022; McClanahan et al., 2023) (84%).

### Preprocessing steps

Preprocessing techniques can be defined as steps taken to prepare raw data into correct format for analysis pipeline or model training. There is a wide range of techniques that are applied depending on core processing. In case of model training, for visual data analysis these include data labelling (for sML and sDL), converting data into tensors, splitting the data into train-val-test datasets, and many more, as shown in Table 4. The first 3 preprocessing steps have been omitted from the analysis below, as these form an obligatory core of preprocessing that computer scientists need to follow to develop AI-based model. In the following section, analysis will focus on the rest of preprocessing techniques, as grouped in Table 4.

**Table 4:** Categories of preprocessing techniques, exemplar techniques utilized in analyzed literature.

Categories of preprocessing techniques	Definition	Exemplar preprocessing techniques
Augmentation	Artificially expand dataset by creating modified copies of the original data.	Flip, Rotation, Multiply, Cutout, Layering near/far sight images
Image preprocessing	Transform raw images into processed images ready for modelling.	Grayscale conversion, binarization, distance transformation, Gaussian blur/filtering
Data normalization / standardization	Scale or align data to reduce variability and ensure comparability.	Min-max normalization, position centering, orientation normalization, contour and scale normalization
Denoising	Remove unwanted elements from the data.	Kalman filter, trajectory smoothing
Image segmentation	In the context of visual data, separating meaningful parts of the data from the noise.	Adaptive thresholding, background subtraction, contour extraction, skeletonization, Region Of Interest (ROI) selection
Dimensionality reduction	Action to reduce the number of features/variables while retaining depth of information from the data.	PCA, MOP, t-SNE
Feature extraction	Deriving structural, temporal, or statistical values from raw or processed data.	Triangulation, Hough circle transformation, morphological operations such as “close”, entropy extraction (BLS), Fuzzy Recurrence Plot (FRP)
Feature encoding	Convert features into structured, model-ready representation.	Transforming keypoints into features, transforming sequences of trajectories into strings, transformation of coordinates to spaciotemporal data

**Figure 11:**
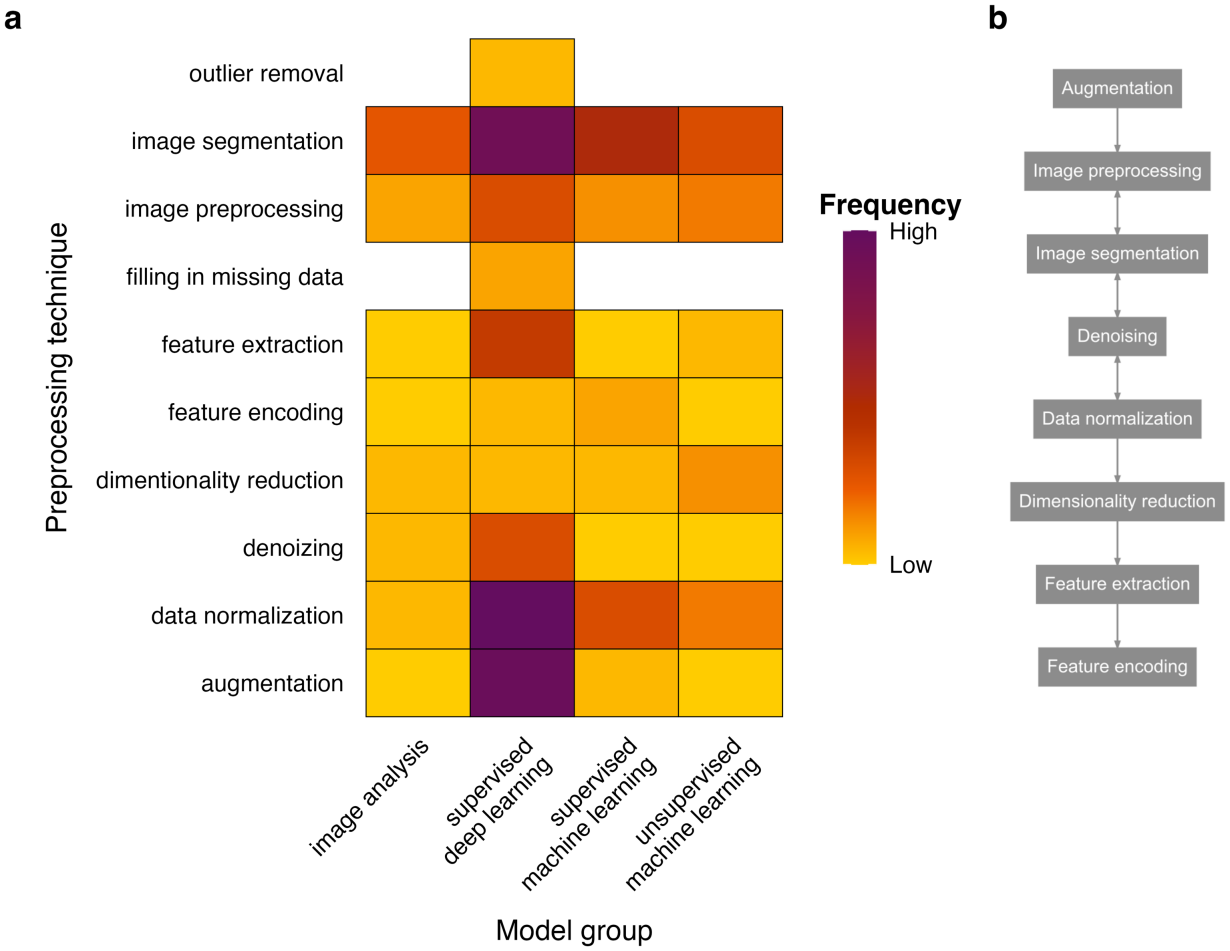
Reported preprocessing techniques according to different model types. Heatmap showing relative proportion of preprocessing techniques reported for each model group **(a)** and exemplary flow diagram of preprocessing steps **(b)**.

In the analyzed literature, there was a high degree of heterogeneity in reporting preprocessing steps. Some papers dedicated the whole subsection to describing steps undertaken to prepare the data for model training, sometimes accompanied by informative diagram; others barely mentioned said techniques. Hence, the data were summarized as a heatmap (Figure 11a) highlighting relative frequencies of preprocessing techniques undertaken according to the model type.

The preliminary step in preprocessing was data augmentation (Stern et al., 2015; Pereira et al., 2019; Graving et al., 2019; Seong et al., 2020; Kasinathan et al., 2020; Karashchuk et al., 2021; Ong et al., 2021; Sharma et al., 2020; Suarez et al., 2021; Saeed et al., 2021; Zhang et al., 2025; Dang et al., 2025; Bates et al., 2022; Galimov and Yakimovich, 2022; Garcia-Garvi et al., 2023; Genaev et al., 2022; Long et al., 2023; Zhang et al., 2024; Jahangir et al., 2023; Majewski et al., 2024; Liu et al., 2024), most frequently used for sDL models (Figure 11). Increasing the dataset size, by images resizing, rotating or reducing image clarity is a common practice in model training, especially for sDL models, which require substantially more input data to achieve high performance. The most used techniques within the image preprocessing category included Gaussian filtering (Hebert et al., 2021; Ahmad et al., 2021; McKenzie-Smith et al., 2025; Lee and Gao, 2023; Ahmad et al., 2024) and grayscale conversion (Kesvarakul et al., 2017; Suarez et al., 2021; Zhan et al., 2025). The former technique blurred the image to prevent overfitting at the same time, putting emphasis on objects of interest. The latter technique allowed to reduce computational cost of image processing. Conversion of images from three-color scale such as RGB format (commonly used in camera recording settings) to single color scale such as greyscale preserves important information while reducing the memory space as well as processing time in model training. Next, many pipelines included image segmentation techniques, such as adaptive thresholding (Girdhar et al., 2015; Hebert et al., 2021; Michels et al., 2019; Bodenstein et al., 2016; Kesvarakul et al., 2017), skeletonization (Javer et al., 2018; Jeong et al., 2019; Layana Castro et al., 2020; Banerjee et al., 2023; Weng et al., 2024; Deng et al., 2024) and background subtraction (Girdhar et al., 2015; Štih et al., 2019; Jeong et al., 2019; Mearns et al., 2020; Michels et al., 2019; Almarinez and Hernandez, 2019; Yang et al., 2025; Weng et al., 2024; Ahmad et al., 2024; Yin et al., 2024) (Figure 11). These techniques also serve the purpose of reducing data depth while preserving important features. Reducing computational cost and unnecessary depth of information optimizes model training time. The denoising and data normalization approaches were also reported in analyzed literature. Normalization included object centering (Gosztolai et al., 2021; Gunes and Gungormus, 2022) and scale normalization (Shamur et al., 2016; Huang et al., 2016; Marques et al., 2018; Mearns et al., 2020; Seong et al., 2020; Gosztolai et al., 2021; Arame et al., 2025; Dang et al., 2025; Keleş et al., 2024; Bian et al., 2019; Galimov and Yakimovich, 2022; Costa et al., 2023; Costa et al., 2022) whereas denoising techniques included trajectory smoothing (Ravan et al., 2023). Dimensionality reduction techniques were rarely reported at preprocessing level (Girdhar et al., 2015; Rudolf et al., 2019; Keleş et al., 2024; Bian et al., 2019). However, many unsupervised machine learning and multi-model pipelines included dimensionality reduction techniques at latter stages of the analysis (Jeong et al., 2019; Mearns et al., 2020; Arame et al., 2025; McKenzie-Smith et al., 2025; Keleş et al., 2024; Gendelev et al., 2024). Finally, feature extraction and feature encoding (Perni et al., 2018; Marques et al., 2018; Javer et al., 2018; Kang et al., 2024; Nguyen et al., 2022; Zhang et al., 2024; Surya et al., 2022) were least commonly reported at this stage (Table 4; Figure 11).

Taken together, reporting of preprocessing steps was not standardized in analyzed literature. Possibly, due to the rapid advancements in AI techniques across a wide range of scientific fields, there is no standardization of methodology reporting. Moreover, many outlined pipelines utilized end-to-end software for tracking pose estimation [e.g., DeepSORT (Si et al., 2022) and DeepLabCut (Karashchuk et al., 2021; Keleş et al., 2024; Manduca et al., 2023)], that already included standard preprocessing steps as part of the model application. Interestingly, over 30% of pipelines utilizing sDL models reported no preprocessing (Aqil et al., 2018; Roosjen et al., 2020; Armalivia et al., 2021; Li et al., 2025; Zhang et al., 2024; Bigge et al., 2024; McClanahan et al., 2023; Widrick et al., 2024) or only data augmentation (Stern et al., 2015; Pereira et al., 2019; Graving et al., 2019; Kukieattikool et al., 2020; Ong et al., 2021; Bentzur et al., 2020; Zhang et al., 2025; Bates et al., 2022; Long et al., 2023; Majewski et al., 2024; Liu et al., 2024) (Supplementary Table S4). Nonetheless, analyzed literature shows a clear preprocessing pipeline as shown in Figure 11b. Data is commonly augmented; images are then resized and color-scale converted followed by denoising and normalization techniques.

### Model architectures

**Figure 12:**
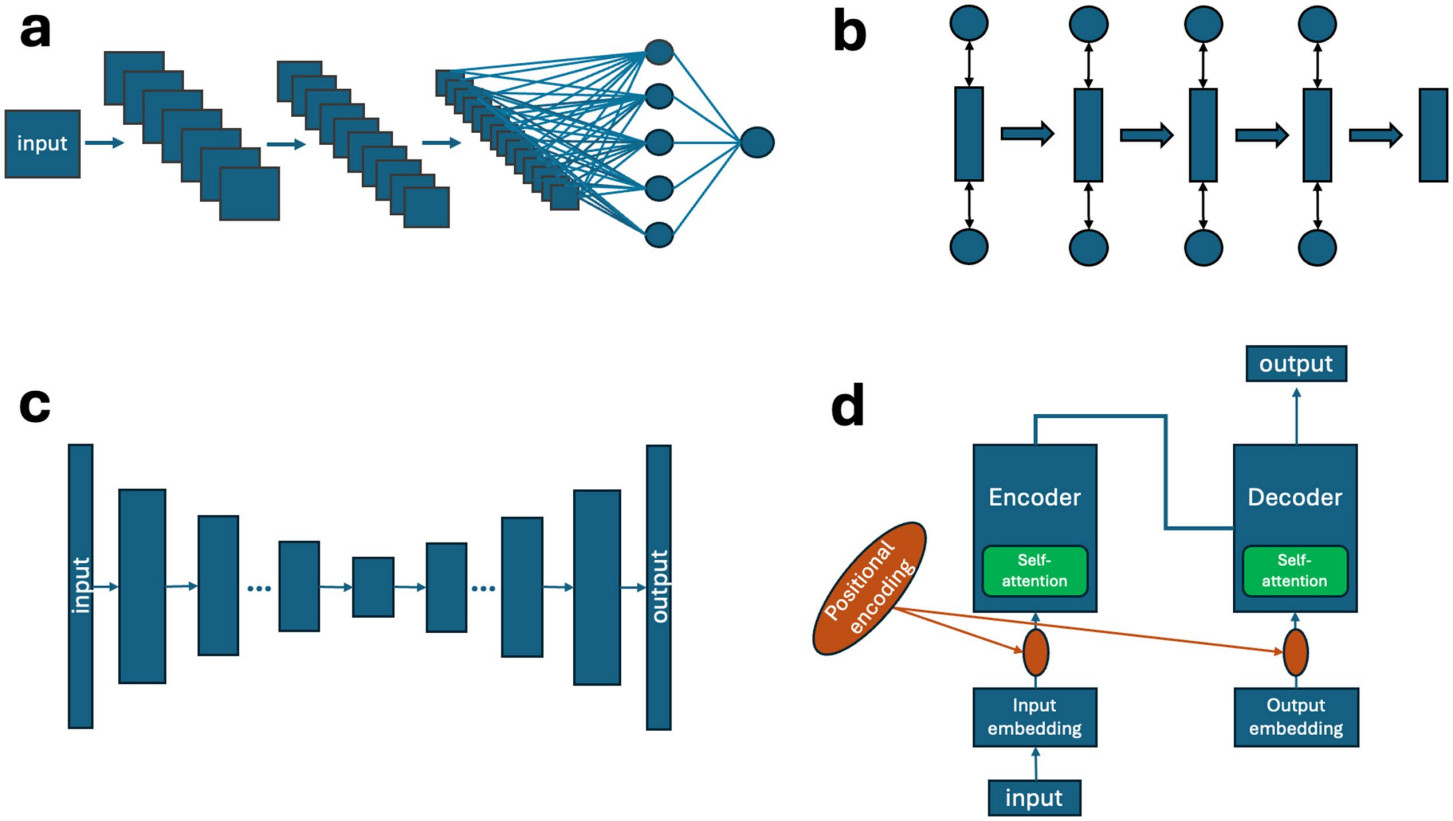
Graphical representation of basic architecture elements of four discussed architecture types: CNN (convolutional neural network) **(a)**, RNN (recurrent neural network) **(b)**, autoencoder **(c)**, transformer **(d)**.

Within a subgroup of deep learning models, the vast majority developed CNN models (85%) (Stern et al., 2015; Mathis et al., 2018; Aqil et al., 2018; Günel et al., 2019; Pereira et al., 2019; Jeong et al., 2019; Jiang et al., 2019; Graving et al., 2019; Roosjen et al., 2020; Kasinathan et al., 2020; Karashchuk et al., 2021; Ong et al., 2021; Gosztolai et al., 2021; Hebert et al., 2021; Ahmad et al., 2021; Bjerge et al., 2021; Sharma et al., 2020; Suarez et al., 2021; Armalivia et al., 2021; Saeed et al., 2021; Gao et al., 2021; Zhang et al., 2025; Zhan et al., 2025; McKenzie-Smith et al., 2025; Bates et al., 2022; Pereira et al., 2022; Ravan et al., 2023; Gore et al., 2023; Keleş et al., 2024; Gendelev et al., 2024; Melis et al., 2024; Zhang et al., 2023; Galimov and Yakimovich, 2022; Obasekore et al., 2023; Garcia-Garvi et al., 2023; Costa et al., 2023; Costa et al., 2022; Manduca et al., 2023; Jang et al., 2024; Genaev et al., 2022; Bigge et al., 2024; McClanahan et al., 2023; Banerjee et al., 2023; Weng et al., 2024; Widrick et al., 2024; Si et al., 2022; Long et al., 2023; Zhang et al., 2024; Zhou et al., 2023; Tiyapunjanit et al., 2023; Surya et al., 2022; Abedin et al., 2023; Jahangir et al., 2023; Javed et al., 2024; Majewski et al., 2024; Gunes and Gungormus, 2022; Nwoko et al., 2023; Liu et al., 2024; Ahmad et al., 2024; Rujito et al., 2023; Yin et al., 2024), followed by Transformers (6%) (Li et al., 2025; Yang et al., 2025; Zhang et al., 2024; Fawzy et al., 2024), RNN (4%), hybrid models of Transformer and CNN architecture elements (3%) (García Garví et al., 2021; Lee and Gao, 2023) and autoencoders (3%) (Seong et al., 2020; Green et al., 2023). These groups have distinct architecture elements highlighted in Figure 12. The characteristics of CNN architectures include convolutional layers for spatial feature extraction and pooling operations for dimensionality reduction (Zhao et al., 2024) (Figure 12a). They are effective for image analysis, especially for static frame-by-frame extractions. On the other hand, RNNs, especially LSTM models, are built from independent blocks that have input and output, with a gating mechanism that discards useless information over time (Mienye et al., 2024; Van Houdt et al., 2020) (Figure 12b). They excel in analyzing temporal information such as motion sequences (Mienye et al., 2024; Van Houdt et al., 2020). Autoencoders are feedforward architectures that compress input to later decompress and reconstruct information (Berahmand et al., 2024) (Figure 12c). They are useful in unsupervised tasks to learn to detect behavioral patterns (Berahmand et al., 2024). Finally, Transformers, being highly diverse in their architecture, can be unified to the following core architecture elements: self-attention module (weights importance of different elements within an input), positional encoding (inputs information on order or special arrangement of an input data) and task-specific head (maps transformed representation into the final output such as classification, detection etc.) (Nerella et al., 2024) (Figure 12d).

**Figure 13:**
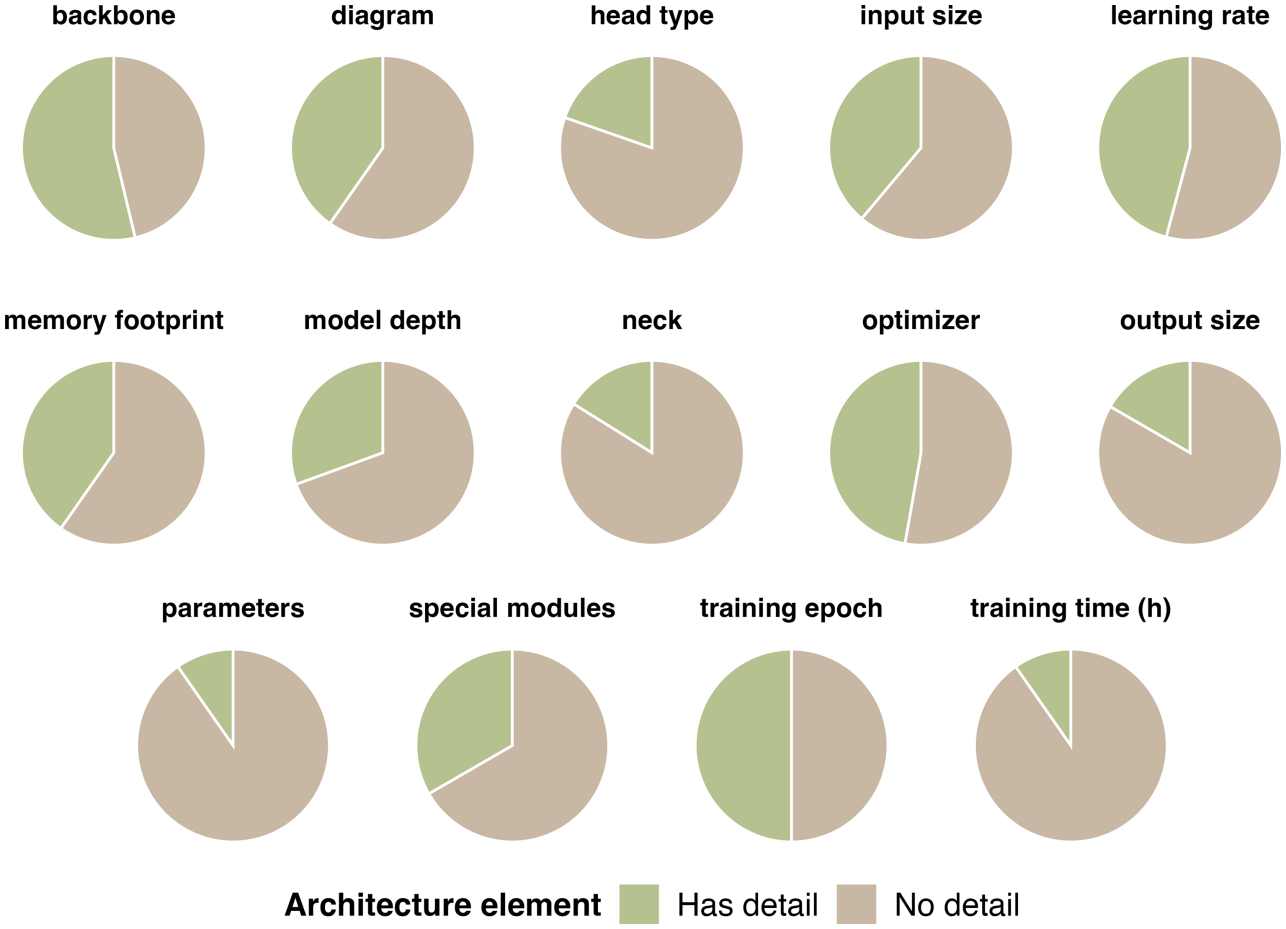
Visual representation of overall reporting of details on model architecture across analyzed literature.

Within analyzed literature, the most commonly reported architecture element was backbone (Mathis et al., 2018; Günel et al., 2019; Pereira et al., 2019; Jiang et al., 2019; Roosjen et al., 2020; Karashchuk et al., 2021; Ong et al., 2021; García Garví et al., 2021; Sharma et al., 2020; Gao et al., 2021; Zhang et al., 2025; Dolata et al., 2025; McKenzie-Smith et al., 2025; Bates et al., 2022; Pereira et al., 2022; Zhang et al., 2023; Galimov and Yakimovich, 2022; Obasekore et al., 2023; Garcia-Garvi et al., 2023; Costa et al., 2022; Zhang et al., 2024; Genaev et al., 2022; McClanahan et al., 2023; Widrick et al., 2024; Long et al., 2023; Zhang et al., 2024; Surya et al., 2022; Jahangir et al., 2023; Javed et al., 2024; Lee and Gao, 2023; Majewski et al., 2024; Gunes and Gungormus, 2022; Nwoko et al., 2023; Liu et al., 2024; Ahmad et al., 2024; Rujito et al., 2023) (Figure 13). Commonly utilized backbones include ResNet50 (Mathis et al., 2018; Pereira et al., 2019; Karashchuk et al., 2021; Sharma et al., 2020; Dolata et al., 2025; Garcia-Garvi et al., 2023; McClanahan et al., 2023; Widrick et al., 2024; Surya et al., 2022; Rujito et al., 2023), CSPDarknet53 (used in YOLO models) (Gao et al., 2021; Zhang et al., 2023; Genaev et al., 2022; Zhang et al., 2024; Javed et al., 2024; Majewski et al., 2024; Nwoko et al., 2023; Liu et al., 2024), and U-Net (McKenzie-Smith et al., 2025; Pereira et al., 2022; Galimov and Yakimovich, 2022; Jahangir et al., 2023) (Supplementary Table S5). In almost 50% of analyzed literature authors reported detailed model training such as type of optimizer used (Günel et al., 2019; Pereira et al., 2019; Graving et al., 2019; Roosjen et al., 2020; Karashchuk et al., 2021; García Garví et al., 2021; Gosztolai et al., 2021; Hebert et al., 2021; Bjerge et al., 2021; Sharma et al., 2020; Suarez et al., 2021; Saeed et al., 2021; Yang et al., 2025; Zhang et al., 2025; Dolata et al., 2025; McKenzie-Smith et al., 2025; Pereira et al., 2022; Ravan et al., 2023; Zhang et al., 2023; Garcia-Garvi et al., 2023; Costa et al., 2023; Zhang et al., 2024; Jang et al., 2024; Pham, 2023; Green et al., 2023; Long et al., 2023; Tiyapunjanit et al., 2023; Surya et al., 2022; Abedin et al., 2023; Lee and Gao, 2023; Deng et al., 2024; Rujito et al., 2023), training epoch (Stern et al., 2015; Günel et al., 2019; Pereira et al., 2019; Jiang et al., 2019; Graving et al., 2019; Roosjen et al., 2020; Kasinathan et al., 2020; Ong et al., 2021; García Garví et al., 2021; Gosztolai et al., 2021; Hebert et al., 2021; Sharma et al., 2020; Suarez et al., 2021; Saeed et al., 2021; Yang et al., 2025; Zhang et al., 2025; Pereira et al., 2022; Ravan et al., 2023; Melis et al., 2024; Zhang et al., 2023; Galimov and Yakimovich, 2022; Obasekore et al., 2023; Garcia-Garvi et al., 2023; Costa et al., 2023; Costa et al., 2022; Zhang et al., 2024; Jang et al., 2024; Pham, 2023; Banerjee et al., 2023; Green et al., 2023; Zhang et al., 2024; Tiyapunjanit et al., 2023; Surya et al., 2022; Abedin et al., 2023; Rujito et al., 2023) and learning rate (Aqil et al., 2018; Günel et al., 2019; Jiang et al., 2019; Graving et al., 2019; Roosjen et al., 2020; Kasinathan et al., 2020; Ong et al., 2021; García Garví et al., 2021; Gosztolai et al., 2021; Hebert et al., 2021; Suarez et al., 2021; Saeed et al., 2021; Yang et al., 2025; Zhang et al., 2025; Dolata et al., 2025; Zhan et al., 2025; Pereira et al., 2022; Ravan et al., 2023; Melis et al., 2024; Zhang et al., 2023; Garcia-Garvi et al., 2023; Costa et al., 2023; Zhang et al., 2024; Jang et al., 2024; Pham, 2023; Genaev et al., 2022; Green et al., 2023; Long et al., 2023; Tiyapunjanit et al., 2023; Liu et al., 2024; Deng et al., 2024) (Supplementary Table S5). The majority of models used Adaptive Moment Estimation (ADAM) optimizer (70%) (Pereira et al., 2019; Graving et al., 2019; Roosjen et al., 2020; Karashchuk et al., 2021; García Garví et al., 2021; Gosztolai et al., 2021; Hebert et al., 2021; Bjerge et al., 2021; Sharma et al., 2020; Suarez et al., 2021; Yang et al., 2025; McKenzie-Smith et al., 2025; Pereira et al., 2022; Ravan et al., 2023; Zhang et al., 2024; Pham, 2023; Long et al., 2023; Tiyapunjanit et al., 2023; Surya et al., 2022; Abedin et al., 2023; Deng et al., 2024; Rujito et al., 2023), 8 papers used Stochastic Gradient Descent (SGD) (24%) (Bjerge et al., 2021; Zhang et al., 2025; Dolata et al., 2025; Zhang et al., 2023; Garcia-Garvi et al., 2023; Costa et al., 2023; Jang et al., 2024; Lee and Gao, 2023) and 2 papers used Root Mean Square Propagation (RMSProp) (6%) (Günel et al., 2019; Saeed et al., 2021). Training epoch ranged from 10 epoch [KAN Transformer (Yang et al., 2025)] to up to 125,000 epoch [Deep autoencoder (Green et al., 2023)]. Similarly, the learning rate of algorithms varied greatly, from 0.1 (Liu et al., 2024) to 0.00001 (Jiang et al., 2019; Jang et al., 2024). Furthermore, size of input data (Stern et al., 2015; Günel et al., 2019; Jiang et al., 2019; Roosjen et al., 2020; Kasinathan et al., 2020; García Garví et al., 2021; Gosztolai et al., 2021; Hebert et al., 2021; Sharma et al., 2020; Saeed et al., 2021; Gao et al., 2021; Yang et al., 2025; Zhang et al., 2025; Zhan et al., 2025; Melis et al., 2024; Kang et al., 2024; Galimov and Yakimovich, 2022; Obasekore et al., 2023; Costa et al., 2023; Genaev et al., 2022; Weng et al., 2024; Zhang et al., 2024; Tiyapunjanit et al., 2023; Deng et al., 2024; Rujito et al., 2023) was more frequently reported than the size of output data (Stern et al., 2015; Günel et al., 2019; Jiang et al., 2019; Roosjen et al., 2020; Kasinathan et al., 2020; García Garví et al., 2021; Gosztolai et al., 2021; Hebert et al., 2021; Sharma et al., 2020; Saeed et al., 2021; Gao et al., 2021; Yang et al., 2025; Zhang et al., 2025; Zhan et al., 2025; Melis et al., 2024; Kang et al., 2024; Galimov and Yakimovich, 2022; Obasekore et al., 2023; Costa et al., 2023; Genaev et al., 2022; Weng et al., 2024; Zhang et al., 2024; Tiyapunjanit et al., 2023; Deng et al., 2024; Rujito et al., 2023) (38% vs. 19%, Figure 13). Memory footprint was reported only in 39% of papers (Mathis et al., 2018; Štih et al., 2019; Günel et al., 2019; Pereira et al., 2019; Jiang et al., 2019; Graving et al., 2019; Gosztolai et al., 2021; Yang et al., 2025; Zhang et al., 2025; Dolata et al., 2025; McKenzie-Smith et al., 2025; Bates et al., 2022; Pereira et al., 2022; Ravan et al., 2023; Gore et al., 2023; Obasekore et al., 2023; Costa et al., 2023; Zhang et al., 2024; Banerjee et al., 2023; Widrick et al., 2024; Long et al., 2023; Zhang et al., 2024; Javed et al., 2024; Lee and Gao, 2023; Majewski et al., 2024; Nwoko et al., 2023; Liu et al., 2024; Ahmad et al., 2024) (out of which in all but 1 (Zhang et al., 2024) as GPU). Model neck was reported only in 16% of papers (Sharma et al., 2020; Dolata et al., 2025; McClanahan et al., 2023; Yin et al., 2024), most commonly as Feature Pyramid Network (FPN) in Mask R-CNN (Sharma et al., 2020; Dolata et al., 2025; McClanahan et al., 2023; Yin et al., 2024) or other R-CNN derivatives (Ahmad et al., 2021; Costa et al., 2022). Finally, the least reported model architecture details was the training time (Mathis et al., 2018; Günel et al., 2019; Kasinathan et al., 2020; Gosztolai et al., 2021; Zhang et al., 2025; Ravan et al., 2023; Gore et al., 2023), reported only in 10% of published research. As seen in Figure 13, the extent of architecture elements reported varies greatly across papers, highlighting an urgent need for standardization of AI-models in research. The raw table with all extracted information of models’ architecture can be reviewed in Supplementary Table S5.

### Evaluation metrics

A key part of model development is the evaluation of its performance. This ensures that model is neither over- nor underfitted, thereby enabling generalization across datasets. It also quantifies predictive accuracy and robustness, providing confidence in model outputs. Evaluation metrics should therefore be reported explicitly and transparently, selected according to the specific task and presented alongside relevant experimental details to enable meaningful interpretation and reproducibility. Within the analyzed literature, 46% of evaluated algorithms reported only 1 metric (Girdhar et al., 2015; Huang et al., 2016; Perni et al., 2018; Javer et al., 2018; Mathis et al., 2018; Aqil et al., 2018; Rudolf et al., 2019; Štih et al., 2019; Günel et al., 2019; Pereira et al., 2019; Graving et al., 2019; Roosjen et al., 2020; Mearns et al., 2020; Seong et al., 2020; Kasinathan et al., 2020; Karashchuk et al., 2021; García Garví et al., 2021; Gosztolai et al., 2021; Hebert et al., 2021; Ahmad et al., 2021; Bjerge et al., 2021; Michels et al., 2019; Bodenstein et al., 2016; Kesvarakul et al., 2017; Almarinez and Hernandez, 2019; Sharma et al., 2020; Arame et al., 2025; Dolata et al., 2025; Zhan et al., 2025; McKenzie-Smith et al., 2025; Pereira et al., 2022; Ravan et al., 2023; Gore et al., 2023; Keleş et al., 2024; Gendelev et al., 2024; Melis et al., 2024; Zhang et al., 2023; Costa et al., 2022; Nguyen et al., 2022; Bigge et al., 2024; Widrick et al., 2024; Fawzy et al., 2024; Majewski et al., 2024; Ahmad et al., 2024; Deng et al., 2024). 19 and 17% of models reported 2 evaluation metrics (Huang et al., 2018; Bornhorst et al., 2019; Wu et al., 2019; Jiang et al., 2019; Saeed et al., 2021; Zhang et al., 2025; McKenzie-Smith et al., 2025; Obasekore et al., 2023; Manduca et al., 2023; Genaev et al., 2022; McClanahan et al., 2023; Banerjee et al., 2023; Widrick et al., 2024; Si et al., 2022; Long et al., 2023; Deng et al., 2024) and 3 (Kukieattikool et al., 2020; Suarez et al., 2021; Gao et al., 2021; Li et al., 2025; Dang et al., 2025; Bates et al., 2022; Bian et al., 2019; Kang et al., 2024; Jang et al., 2024; Zhang et al., 2024; Tiyapunjanit et al., 2023; Abedin et al., 2023; Lee and Gao, 2023; Nwoko et al., 2023; Liu et al., 2024; Yin et al., 2024) respectively, and the remaining 18% reported 4 or more metrics (Shamur et al., 2016; Ong et al., 2021; Armalivia et al., 2021; Yang et al., 2025; Galimov and Yakimovich, 2022; Garcia-Garvi et al., 2023; Costa et al., 2023; Zhang et al., 2024; Pham, 2023; Weng et al., 2024; Green et al., 2023; Zhou et al., 2023; Surya et al., 2022; Jahangir et al., 2023; Javed et al., 2024; Gunes and Gungormus, 2022; Rujito et al., 2023) (Supplementary Table S6).

**Figure 14:**
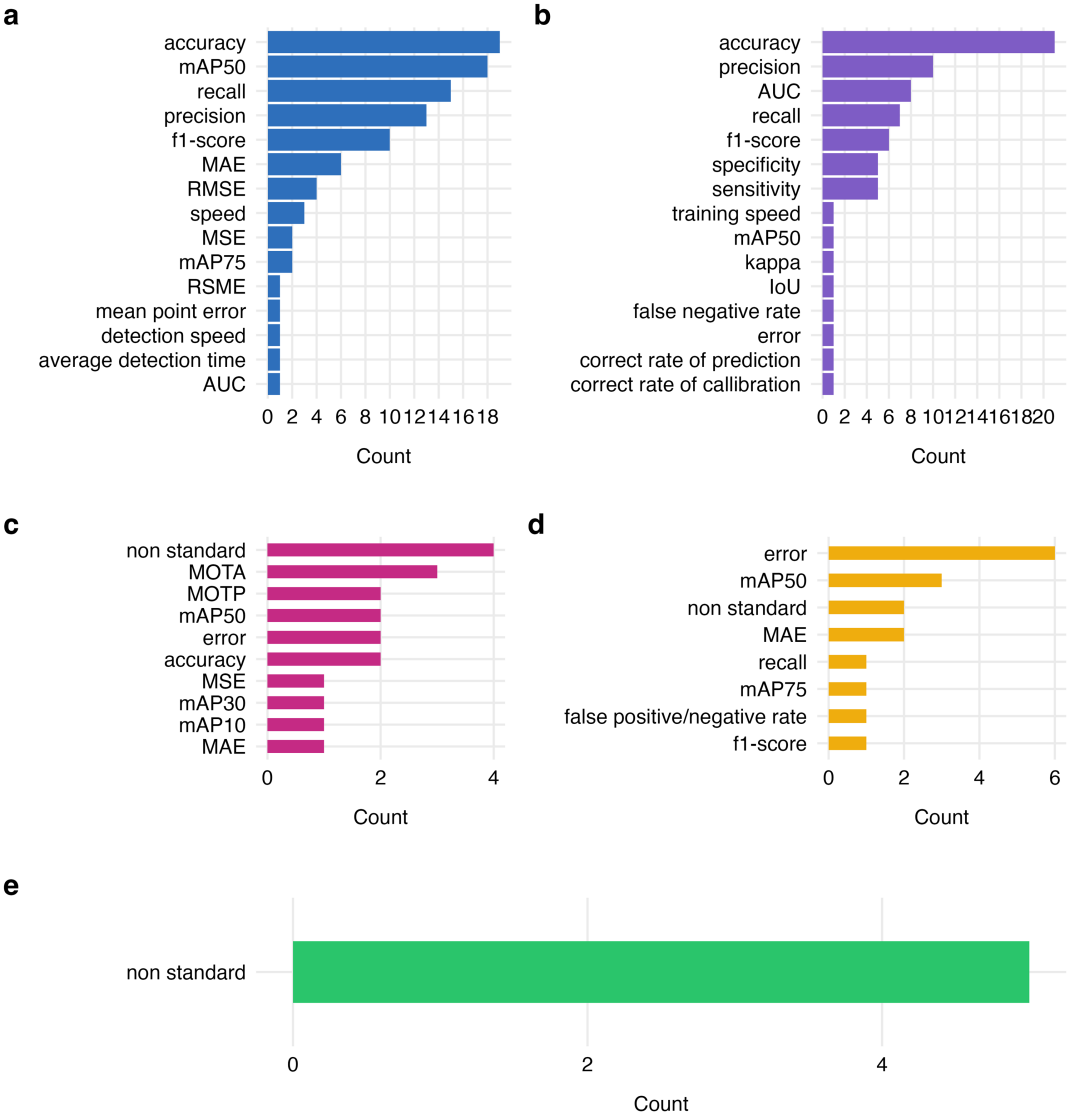
Overview of evaluation metrics reported for model task’s: detection, incl. detection and counting **(a)**, classification **(b)**, tracking **(c)**, pose estimation **(d)**, and clustering and dimensionality reductions **(e)**. mAP50; mean average precision at an intersection over union threshold of 0.5, MAE, mean average error; AUC, area under the curve; RMSE, root mean square error; MOTA, multi-object tracking accuracy; mAP75, mean average precision at an intersection over union threshold of 0.75; MSE, mean standard error; MOTP, multi-object tracking precision.

Figure 14 shows the frequency of reported evaluation metrics across different model tasks: detection, classification, tracking, pose estimation and clustering. For detection tasks the most frequently used metrics were accuracy (Seong et al., 2020; Ong et al., 2021; Ahmad et al., 2021; Kesvarakul et al., 2017; Armalivia et al., 2021; Gao et al., 2021; Dang et al., 2025; Zhan et al., 2025; Bian et al., 2019; Obasekore et al., 2023; Jang et al., 2024; Genaev et al., 2022; Zhou et al., 2023; Jahangir et al., 2023; Javed et al., 2024; Gunes and Gungormus, 2022; Ahmad et al., 2024) and mean average precision measured at the 50% intersection over union threshold (mAP50), reported 19 and 18 times, respectively (Armalivia et al., 2021; Gao et al., 2021; Zhang et al., 2025; Dolata et al., 2025; Zhang et al., 2023; Garcia-Garvi et al., 2023; Costa et al., 2022; Zhang et al., 2024; Genaev et al., 2022; Bigge et al., 2024; Weng et al., 2024; Zhang et al., 2024; Javed et al., 2024; Lee and Gao, 2023; Fawzy et al., 2024; Nwoko et al., 2023; Liu et al., 2024; Deng et al., 2024). Recall, precision and f1-score have also been reported frequently, respectively in 15 (Ong et al., 2021; Armalivia et al., 2021; Garcia-Garvi et al., 2023; Costa et al., 2023; Zhang et al., 2024; Weng et al., 2024; Zhang et al., 2024; Zhou et al., 2023; Jahangir et al., 2023; Javed et al., 2024; Lee and Gao, 2023; Gunes and Gungormus, 2022; Nwoko et al., 2023; Liu et al., 2024; Yin et al., 2024), 13 (Ong et al., 2021; Obasekore et al., 2023; Garcia-Garvi et al., 2023; Costa et al., 2023; Zhang et al., 2024; Weng et al., 2024; Zhang et al., 2024; Jahangir et al., 2023; Javed et al., 2024; Gunes and Gungormus, 2022; Nwoko et al., 2023; Liu et al., 2024; Yin et al., 2024) and 10 (Ong et al., 2021; Armalivia et al., 2021; Garcia-Garvi et al., 2023; Costa et al., 2023; Zhang et al., 2024; Weng et al., 2024; Jahangir et al., 2023; Majewski et al., 2024; Gunes and Gungormus, 2022; Yin et al., 2024) model evaluations (Figure 14a). Other metrics reported included common regression metrics (MAE, RMSE, MSE), metrics related to detection speed, and least frequently area under the ROC curve (AUC) (Figure 14a). For classification tasks, the most commonly reported metric was, as in detection, accuracy (Shamur et al., 2016; Javer et al., 2018; Aqil et al., 2018; Jeong et al., 2019; Kasinathan et al., 2020; García Garví et al., 2021; Almarinez and Hernandez, 2019; Suarez et al., 2021; Saeed et al., 2021; Arame et al., 2025; Galimov and Yakimovich, 2022; Manduca et al., 2023; Pham, 2023; Nguyen et al., 2022; McClanahan et al., 2023; Widrick et al., 2024; Tiyapunjanit et al., 2023; Surya et al., 2022; Abedin et al., 2023; Rujito et al., 2023), appearing in 21 evaluations, followed by precision (Kukieattikool et al., 2020; Suarez et al., 2021; Saeed et al., 2021; Kang et al., 2024; Galimov and Yakimovich, 2022; Pham, 2023; Green et al., 2023; Surya et al., 2022; Abedin et al., 2023; Rujito et al., 2023), AUC (Shamur et al., 2016; Gendelev et al., 2024; Galimov and Yakimovich, 2022; Manduca et al., 2023; Pham, 2023; Green et al., 2023; Widrick et al., 2024), recall (Kukieattikool et al., 2020; Suarez et al., 2021; Kang et al., 2024; Galimov and Yakimovich, 2022; Surya et al., 2022; Abedin et al., 2023; Rujito et al., 2023), f1-score (Kukieattikool et al., 2020; Bjerge et al., 2021; Kang et al., 2024; Pham, 2023; Surya et al., 2022; Rujito et al., 2023). Specificity and sensitivity metrics were also reported in 5 evaluations (Shamur et al., 2016; Bornhorst et al., 2019; Pham, 2023; Green et al., 2023; Tiyapunjanit et al., 2023) (Figure 14b). Tracking tasks were most frequently assessed using non-standard metrics such as comparison to other tracking platforms or manual tracking (Perni et al., 2018; Štih et al., 2019; Banerjee et al., 2020; Melis et al., 2024), multi-object tracking accuracy (MOTA) and multi-object tracking precision (MOTP) were also frequently reported (Yang et al., 2025; Si et al., 2022; Deng et al., 2024). Mean average precision (at IoU 50, 30 and 10), accuracy, error, MSE and MAE metrics were also reported (Figure 14c). Evaluation of pose estimation models was most commonly reported using mean or average error (Günel et al., 2019; Pereira et al., 2019; Gosztolai et al., 2021; McKenzie-Smith et al., 2025; Ravan et al., 2023; Gore et al., 2023), and less frequently reported using mAP50, non-standard evaluations via direct comparison to other pose estimation platforms, MAE, recall, false positive to negative rate, and f1-score (Figure 14d). Finally, due to inherently different nature of unsupervised learning methods, including clustering and dimensionality reductions, models based on these techniques were evaluated using non-standard metrics such as graphical representations (Huang et al., 2016; Rudolf et al., 2019; Mearns et al., 2020) or statistical testing (Girdhar et al., 2015; McKenzie-Smith et al., 2025). As seen in Figure 14 and in supporting materials (Supplementary Table S6), there is a high heterogeneity of reported evaluation metrics. Its direct consequences will be further discussed in the discussion section.

## Discussion

Invertebrate model organisms such as *C. elegans* or *D. melanogaster* remain among the most widely used systems in biology and biomedical research (Kimble and Nüsslein-Volhard, 2022). Their physiology, behavior, and genetics are well established through decades of investigation (Kimble and Nüsslein-Volhard, 2022; Pastorino et al., 2024), providing a robust foundation for experimental studies. These organisms, along with other invertebrates and an increasing number of larval models, offer a distinct advantage over vertebrates such as mice or rats in the context of high-throughput screenings and early-stage research (Pastorino et al., 2024). Behavioral analysis is an emerging, powerful approach in those types of studies, enabling the systematic assessment of functional outcomes that correlate organism-level phenotypes with molecular and cellular mechanisms, and phenomena (Adjé et al., 2023). With the rapid adoption of AI-based methods by the scientific community, the potential of behavioral analysis in these model organisms has become a subject of active investigation.

The growing interest is reflected in a steady rise in publications over the last 15 years (Figure 2). Fields such as neuroscience, epidemiology, and agriculture (Figure 6) started to adopt AI-based methods to study behavior, using over 15 different model organisms (Figure 5). In the last decades authors from over 34 countries shared their research in conference proceedings or peer-reviewed journals (Figure 3), out of which 74% was published by journals with impact factor of 3 and more (Figure 4). This global distribution of research highlights both the widespread adoption and growing scientific maturity of AI-driven behavioral analysis.

Importantly, this expansion is not limited to behavioral studies but reflects a broader transformation of how research is conducted across disciplines. AI-enabled approaches are reshaping how scientific questions are formulated and what insights are extracted from the data. For instance, in toxicology machine and deep learning supports the development of biosensors and screening platforms, enabling faster assessment of chemical toxicity (Jeong et al., 2019; Bodenstein et al., 2016; Gendelev et al., 2024). In agriculture and ecology, AI-enabled tools support rapid and accurate species identification and counting, accelerating biodiversity assessment and preventing pests’ infestation hence increasing crop yields (Roosjen et al., 2020; Bjerge et al., 2021; Suarez et al., 2021; Arame et al., 2025; Obasekore et al., 2023; Gunes and Gungormus, 2022). On the other hand, in neuroscience and genetics, algorithms facilitate deeper insights into organisms’ complex behavior (Javer et al., 2018; Zhan et al., 2025; Pham, 2023; Nguyen et al., 2022) and ultimately accumulate our understanding of these organisms’ biology. Hence, the expansion of AI-enabled methods across disciplines automates previously labor-intensive and error-prone tasks, facilitates new discoveries and as a result opens up the space to ask new research questions and look at the behavior from a rediscovered perspective.

When formulating a research question in behavioral analysis, it is essential to clearly define behavioral characteristics of interest and as a result select an appropriate model for the given task. Model tasks can be broadly categorized into three groups based on their approach to quantify behavioral traits. First, detection and classification algorithms, often coupled with tracking module, categorize behaviors based on predefined criteria such as locomotion parameters (e.g., distance traveled, speed) or morphological features (e.g., body curvature, coiling). These approaches have traditionally relied on supervised machine learning models such as RF, SVM, or KNN, valued for their relatively low computational demands and high interpretability (Uddin and Lu, 2024). sDL models, such as YOLO or EfficientNet, have also found applications in these tasks enabling analysis of more complex and unstructured data, at the expense of requirement for larger computational power and training data resources (Talaei Khoei et al., 2023). These supervised learning approaches, regardless of their computational efficiency and interpretability, depend on researcher-defined categories treating behavior as quantifiable events, which may overlook ambiguous behaviors or temporal patterns. A second type of approach to analysis of behavioral data are algorithms utilizing pose estimation and tracking to extract keypoints (e.g., head, limb or body coordinates) and describe movement kinematics. Such models are inherently objective, capturing raw motion data without imposing predefined behavioral labels, leaving data interpretation to the scientists. They have been used to quantify fine-scale phenotypes such as *D. melanogaster* body kinematics (Karashchuk et al., 2021) or behavioral changes of Mediterranean fruit fly exposed to minimal doses of intoxicants (Manduca et al., 2023). These models, as opposed to detection or classification algorithms, transform behavioral analysis from binary towards real-time behavior parametrization and quantification. Moreover, pose estimation algorithms enable discoveries of previously unknown behavioral readouts, such as microbehavior called “haltere switch” indicating deeper sleep in *D. melanogaster* (Keleş et al., 2024), enriching pool of behavioral parameters for more comprehensive and complex analysis. A third approach to study behavior is use of unsupervised learning. Algorithm, with no predefined conditions, can objectively structure given data via clustering and dimensionality reduction methods. This enables the discovery of novel behavioral motifs, such as swimming patterns (Marques et al., 2018) or prey-capture swim-bouts (Mearns et al., 2020) in *D. rerio* larvae. These data-driven approaches minimize bias from predefined labels and are highly exploratory, but they pose significant challenges for biological interpretation and require careful validation.

**Figure 15:**
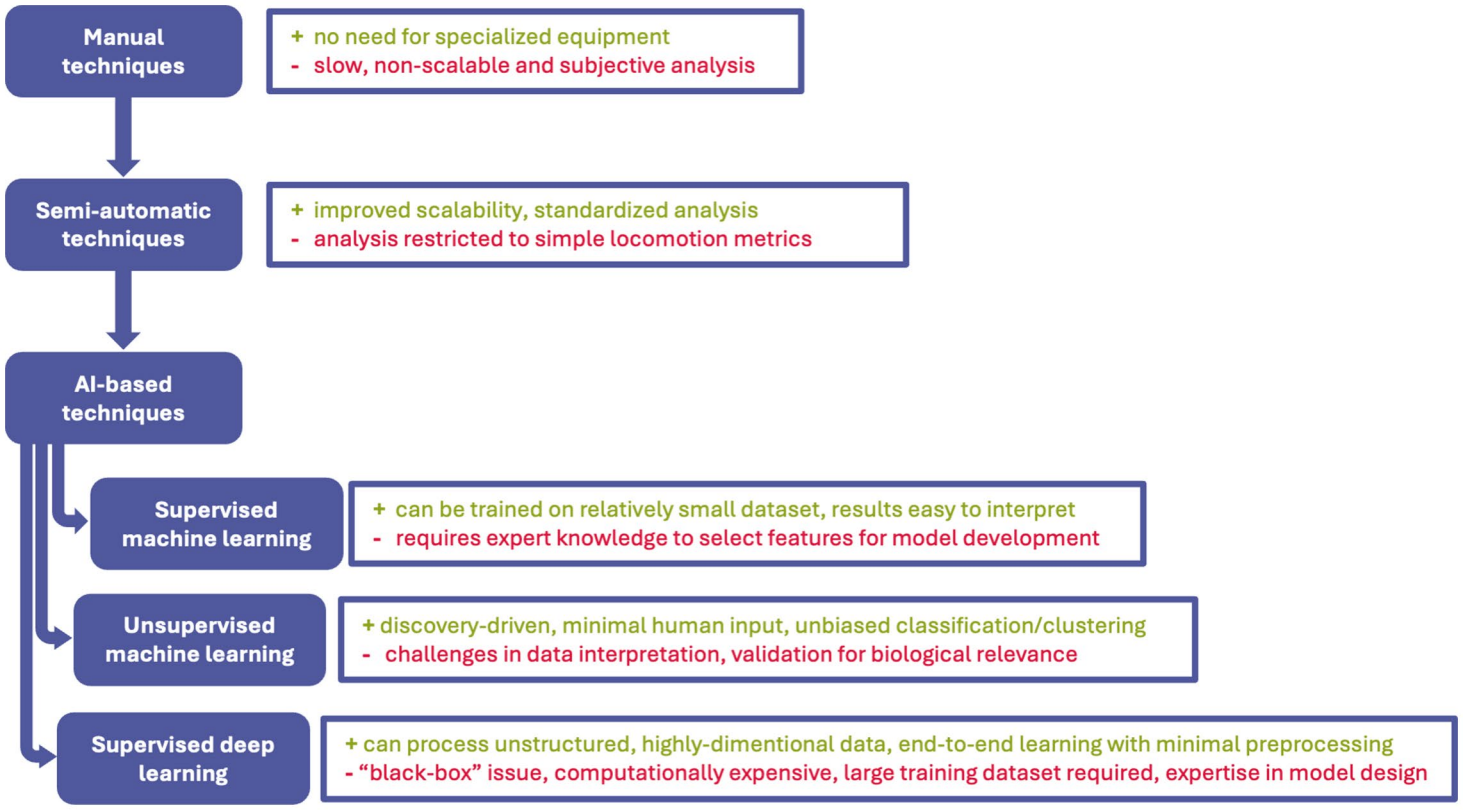
Advantages (colored green) and disadvantages (colored red) of methods used in behavioral analysis.

The choice of AI-based approach should be guided by how behavior is going to be quantified and interpreted. With the growing diversity of available methods, it is important to consider the trade-offs between them, as summarized in Figure 15. Historically, behavioral analysis was based on labor- and time-intensive manual observations. These studies did not require specialized equipment and led to biologically relevant interpretation of behavior; however, they suffered from low efficiency and observer bias. The semi-automatic techniques improved scalability and enabled standardized analysis, contributing to reduction in observer bias. Nonetheless, these techniques reduced behavior to simple locomotive parameters. The emergence of AI-based approaches had addressed many of these limitations, enabling more detailed and scalable analysis, but had also introduced new challenges important to consider when choosing a model for behavioral analysis. sML, used for classification and less frequently for detection and tracking (Figure 8), has proven particularly useful in analyses of smaller datasets, especially when predicting binary outcomes (Uddin and Lu, 2024). However, the main limitation lies in their low performance when it comes to learning complex and nonlinear behavioral patterns. Compared to sML, sDL can process highly dimensional, unstructured data and yield more detailed interpretations (Alloghani et al., 2020). For instance, pose estimation allowed detection of stereotyped behavior of *D. melanogaster* and enabled analysis of behavioral variation over time, capturing microbehaviors that were previously difficult to quantify using conventional methods (McKenzie-Smith et al., 2025). However, these models are typically less explainable. This limitation, referred to as “the black box” problem, is the direct consequence of deep learning models’ architecture. For example, in CNN models, most used in behavioral analysis, the multiple convolutional layers and successive data transformations across layers make the training process difficult to explain (Talaei Khoei et al., 2023). While sDL provides clear advantages for complex tasks such as pose estimation and tracking, for simpler tasks, such as detection or classification, the trade-offs between sDL and sML should be carefully considered. A distinct approach to study behavior is unsupervised machine learning, utilized for clustering and dimensionality reduction tasks (Figure 8). An advantage of these approaches is unbiased exploration and potential to discover novel behavioral patterns. However, this comes at the expense of model complexity and challenges with placing and evaluating the findings in the biologically relevant context (Alloghani et al., 2020). For instance, based on domain knowledge and classification of basic kinematic parameters, eleven behavioral models in *C. intestinalis* larvae had been identified using agglomerative clustering (Rudolf et al., 2019). As a result, uML approaches require interdisciplinary computational and biological expertise and are often coupled with additional analysis in the form of supervised learning or statistical analysis. Some research papers proposed combining unsupervised approaches with supervised machine learning (Rudolf et al., 2019) or supervised deep learning (Jeong et al., 2019) to cluster behavioral patterns and later classify the behavior. Other developed complex pipelines involving tracking or pose estimation (via sDL), combined with classification and clustering or dimensionality reduction techniques (Keleş et al., 2024; Gendelev et al., 2024; McKenzie-Smith et al., 2025). These models are rarely implemented, possibly due to their complexity and requirement for interdisciplinary collaboration. Their potential at the interface of biology and computational science remains to be discovered.

The analysis of published literature indicates that since 2021, sDL models started to dominate the landscape of behavioral analysis in invertebrates and larval model organisms (Figure 7A). This trend can be attributed to several converging factors. First, the increasing availability of GPU computing has enabled the scaling of deep learning architectures, allowing them to be trained and applied to large and complex datasets. Second, the emergence of user-friendly frameworks such as YOLO (Redmon et al., 2015) has lowered the technical barrier for adoption, providing researchers without extensive computational expertise with accessible tools for detection, classification, and tracking. For example, YOLO hosted on Roboflow offers an intuitive online interface where users can upload and annotate images and train models with minimal coding requirements (Tomassini et al., 2024). Finally, the development of open-source platforms for pose estimation such as DeepLabCut (Mathis et al., 2018) and SLEAP (Pereira et al., 2022) has provided flexible tools for complex pose estimation and behavioral analyses. The impact of such platforms is evident in the widespread use of DeepLabCut, whose original paper, published in 2020 (Mathis et al., 2018), has been cited more than 4,300 times (Nature, sampled on 14/10/2025), underscoring its role in enabling reproducible research across diverse model organisms and experimental conditions. The shift from classical machine learning towards supervised deep learning models is evident from described literature. However, it remains unclear whether this transition is primary driven by the need to capture increasingly complex behavioral patterns or by the growing accessibility of deep learning tools, with often present low technical and computational barriers to use. In this context, despite the widespread availability of AI-based methods, it is essential that model selection and experimental approaches remain guided by the underlying research question rather than by methodological convenience.

Within the deep learning algorithms there has been an emergence of several subgroups worth mentioning due to their potential in behavioral research. Most dominant sDL-based group, CNN models, are characterized by high efficiency and automation of the analysis process, especially in the context of visual data processing (Zhao et al., 2024). However, their limitation is their inability to process temporal dependencies, which limits them in motion sequence prediction tasks (Zhao et al., 2024). One explored approach to combat this limitation is the integration of optical flow to improve apparent motion patterns in the images (Manduca et al., 2025). Another group of sDL models, sequential architectures, include RNN and transformers. They are increasingly being used to enable the analysis of dynamic changes over time. RNN models, despite their simple architecture, work well for trajectory prediction and motion micro-pattern detection (Mienye et al., 2024). Transformers, on the other hand, allow for the analysis of complex spatiotemporal contexts, making them particularly useful for tasks involving the recognition of intentional behaviors and motivational states (Nerella et al., 2024). Despite highlighted advantages of both RNN and Transformer architectures, they are only emerging in the field and comparative studies. A between different architectures across model tasks will reveal their advantages and disadvantages in behavioral analysis of invertebrate and larval model organisms.

Most models used in behavioral analysis fall under category of supervised learning (Figures 7a, 8), meaning they require labeled data for training (Alloghani et al., 2020). This approach is time-consuming and it relies on expert annotations. Moreover, dependence on human labeling introduces bias, as models learn from the subjective assumptions of researchers, which can limit their ability to capture unprecedented behavioral patterns. One approach to reducing this limitation is the incorporation of active learning modules. Pereira et al. developed a CNN-based pose estimation model that, alongside pose estimation, identifies uncertain or novel poses and enables users to manually annotate identified images via graphical user interface (Pereira et al., 2019). Gunel et al. incorporated a module that iteratively reprojects 3D poses to detect and correct 2D errors, allowing further training without user intervention (Günel et al., 2019). Both approaches show how models can constantly improve performance, while expanding their capabilities to capture the broader set of behavioral patterns (Pereira et al., 2019). Implementing such strategies in the study of complex and dynamic behaviors may enhance model adaptability across organisms and experimental conditions. Another complementary approach is transfer learning, which has been applied to both pose estimation tasks (Mathis et al., 2018; Karashchuk et al., 2021) and classification tasks (Aqil et al., 2018; Tiyapunjanit et al., 2023). By leveraging previously learned patterns, transfer learning accelerates adoption to new datasets and facilitates application across different model organism and behavioral contexts.

Nonetheless, with increased automation of supervised learning model preparation, the danger of overreliance on AI becomes even more prominent. While the growing number of AI-based tools lowers the barrier for conducting computational analysis, it remains critical that discoveries are biology-driven. In this context, the quality of input data becomes a central determinant of model performance and interpretability. High-quality, well-annotated, and representative datasets are essential to ensure that models capture biologically meaningful patterns rather than artifacts or biases introduced during data collection. However, datasets remain highly imbalanced, with frequent locomotor patterns overrepresented while rare but biologically important behaviors can be undetected or entirely missed (Figures 9, 10). Developing behavioral ontologies and open, standardized datasets could help overcome this issue by enabling the integration of learned behavioral patterns across studies, similar to the role of ImageNet or COCO in computer vision. Reporting datasets, as demonstrated by Li et al. (2025) and Pereira et al. (2022), is an important step towards transparency and forms foundation for future datasets integration to group behavior and classify behavioral patterns across different model organisms. Poor data quality or inconsistent annotation can propagate errors throughout the analysis pipeline, ultimately limiting the reliability and generalizability of findings.

Building on the need for standardized and transparent datasets, clear and consistent reporting practices are equally critical for ensuring reproducibility. Reporting should include comprehensive documentation of experimental design, data collection and processing, model choice and architecture, and the use of well-defined evaluation metrics. Such standards not only improve reproducibility but also facilitate comparison across studies and support the integration of findings within the field. Currently, AI-based behavioral studies of invertebrate and larval organisms lack such standardization. Key experimental details, such as recording parameters including fps, resolution and lightning conditions, are often insufficiently reported, despite their direct impact on preprocessing, model training, and performance. Without this information, reproducing results or reusing models becomes challenging, as these conditions often need to be closely matched.

In parallel, model architecture is reported in an unstructured and fragmented manner (Figure 13). The lack of transparency is particularly problematic in deep learning approaches, where complex model structures can limit interpretability. Without sufficient understanding of model architecture, it becomes difficult to assess the validity and reliability of the findings. As a result, studies may either be questioned or become difficult to build upon, ultimately hindering scientific progress.

Similarly, evaluation metrics should reflect the model’s task and enable cross-study comparison. While predictive performance, in majority of analyzed studies, is reported by at least 1 metric (Figure 14), the use of different metric makes direct comparison across studies difficult. Moreover, as shown on Figure 14, there is a substantial heterogeneity in reported evaluation metrics, raising the question of whether they are consciously selected to best reflect model performance. For detection tasks, recommended metrics include intersection over union (IoU) and measures derived from true/false positives and negatives ratios (incl. precision, recall, f1-score, accuracy, ROC AUC). However, metric selection should also depend on the model type (sDL vs. sML) and the number of objects being detected, with IoU being the primary evaluation metric in single-object detection. Similarly, classification tasks, also depending on number of classes, should be evaluated using appropriate metrics. In this case, the extensive methodological background, especially for sML, may explain greater alignment between recommended and reported metrics (incl. accuracy, precision, f1-score, recall, AUC, sensitivity, and specificity).

In contract, tracking and pose estimation tasks exhibit even greater heterogeneity in evaluation practices. MOTA and MOTP, alongside classification-based metrics suggest that tracking performance is often assessed through detection accuracy. For pose estimation, error-based metrics are commonly used to quantify discrepancies between actual and predicted key-points positions. As pose estimation emerges as a new and rapidly developing AI-enabled tool in behavioral analysis, there is a growing need for clearer guidelines on the selection of appropriate evaluation metrics. The observed inconsistencies hider reproducibility and limit direct comparison across models. The limited number of comparative studies that benchmark different approaches is likely a direct consequence of this fragmentation.

**Figure 16:**
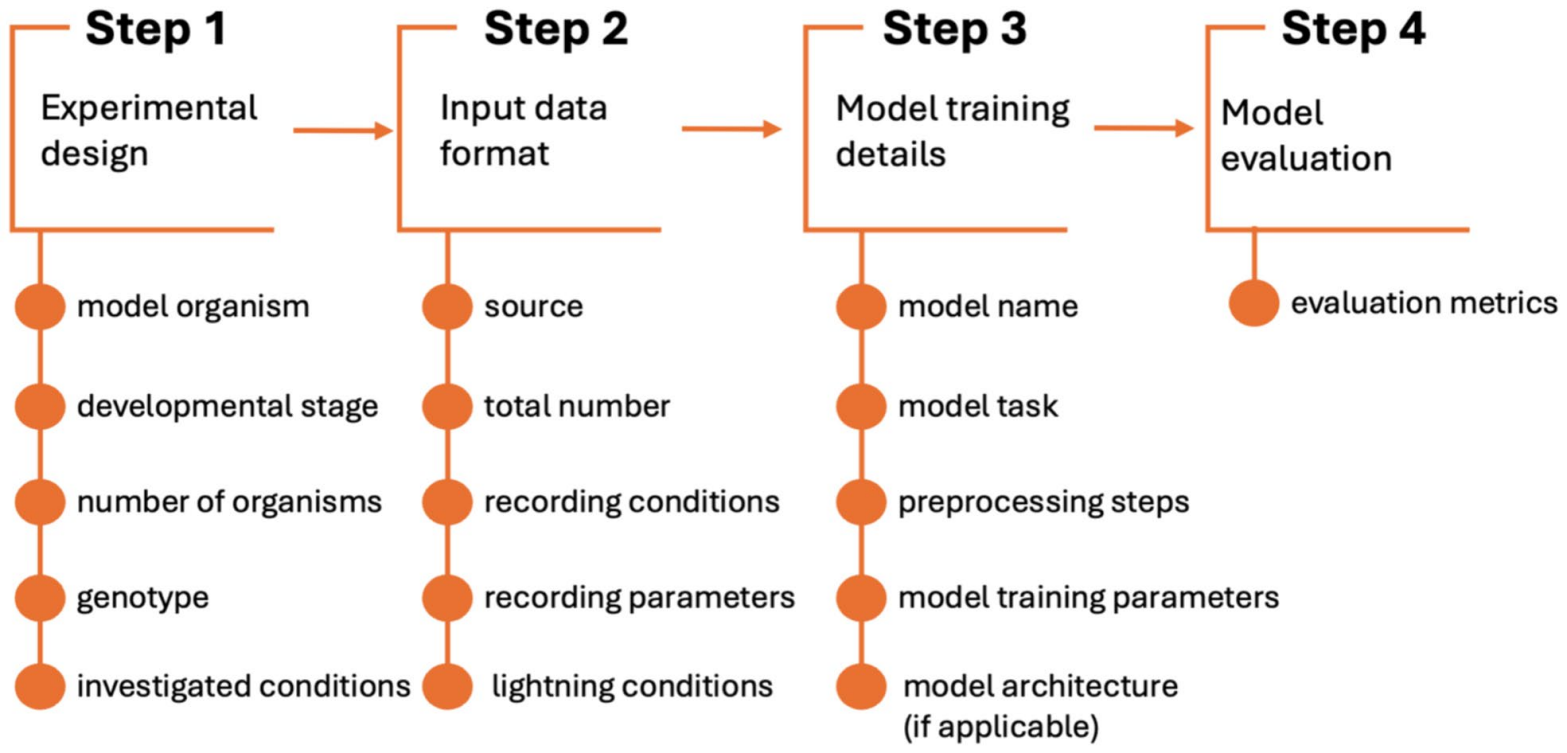
Proposed workflow for recording key information on model design and evaluation for behavioral studies on invertebrate and larval model organisms.

To address these challenges, we propose the introduction of a standardized reporting template for behavioral studies (Supplementary File S1). Such framework should include detailed documentation of experimental design, input data, preprocessing steps, model training details, and evaluation metrics, thereby improve transparency and reproducibility, and ultimately facilitate cross-laboratory comparison and cumulative progress in the field. The proposed workflow for reporting these elements can be reviewed in Figure 16, and complete reporting data sheet can be viewed in Supplementary File S1.

Looking ahead, behavior is increasingly recognized as a powerful and versatile readout across scientific disciplines, from deconstructing animal actions to enabling high-throughput toxicological screens and early-stage drug discovery. As complex and multidimensional aspect of physiology, it offers substant potential for quantitative and scalable analysis. Advances in computational science and AI-based methods have not only increased throughput but have fundamentally transformed how behavior can be studied, moving beyond observer-defined categories towards multidimensional phenotypes. These approaches enable precise pose estimation, tracking, and behavioral classification, providing powerful tools for both large-scale and micro-movement behaviors. They have also enabled discoveries of new behavioral traits through unsupervised learning approaches.

The field of AI-based behavioral analysis of invertebrates and larval model organisms is rapidly evolving. Notably, during the revision process eight original papers had been published further exploring combination of UL and ML (Hageter et al., 2025), high-throughput screening platforms (Van Camp et al., 2025; Shappell et al., 2025; Mullen et al., 2026) and improved method to tackle problems with identifying and tracking overlapping worms (Weheliye et al., 2025). Maintaining awareness of these developments is important for future models development and their integration into research practice.

At the same time, effective use of these methods required a biology-driven framework, in which experimental design, model selection and interpretation are guided by the underlying biological question. As AI becomes increasingly integrated into scientific workflows, it is therefore essential to avoid overreliance on automated outputs and instead apply these tools selectively, ensuring that analyses remain interpretable, biologically meaningful and aligned with the experimental context. Standardized reporting practices will be essential to enable collaboration and cross-disciplinary progress. Continued development of AI-based behavioral analysis holds significant potential to advance toxicology, pharmacology, and fundamental research, driving a deeper understanding of animal behavior across model systems.

## Data Availability

The datasets presented in this study can be found in online repositories. The names of the repository/repositories and accession number(s) can be found in the article/Supplementary material.
